# Supplementary material for: Integral approach to biomacromolecular structure by analytical-ultracentrifugation and small-angle scattering
Source: Commun Biol. 2020 Jun 8;3:294. doi: 10.1038/s42003-020-1011-4 (PMC7280208; doi:10.1038/s42003-020-1011-4)
Supplement: Supplementary file 1 — Supplementary Information [file 42003_2020_1011_MOESM1_ESM.pdf]

SUPPLEMENTARY FIGURES

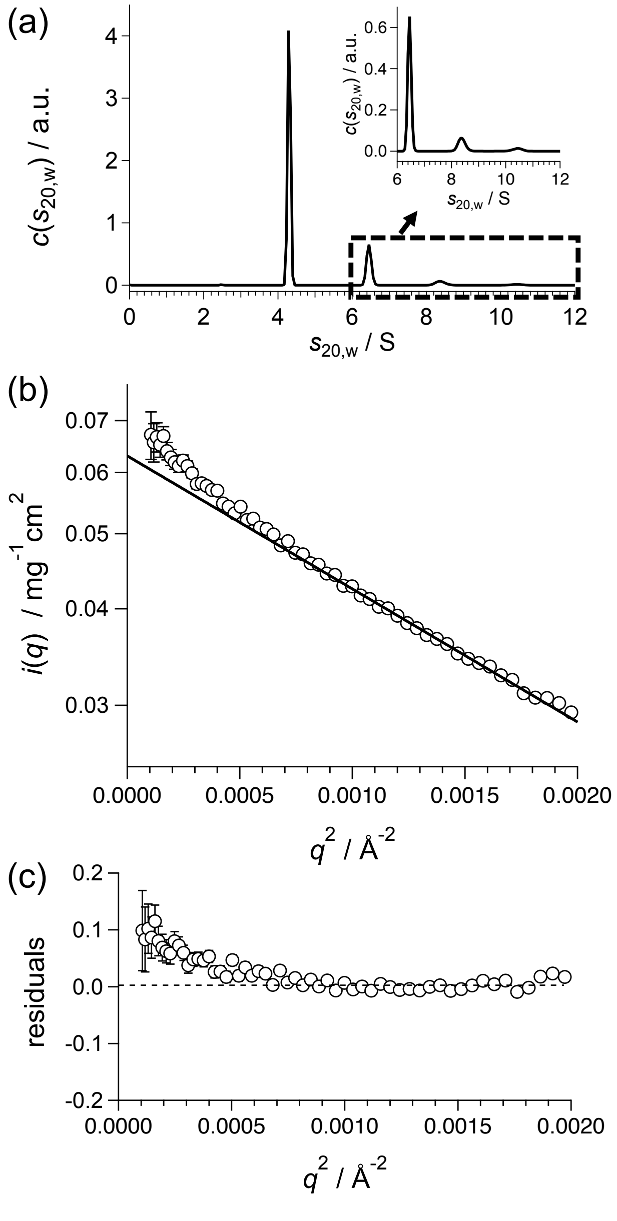

Supplementary Fig.1. Typical example of AUC and SAS intensity including a large amount of aggregates, which makes the upturn of SAS intensity and deviation from Guinier approximation in the low q-range. The sample was a solution without any purification after dissolving the purchased BSA in 100mM Tris/HCl (pH7.5) buffer containing 100mM NaCl (mass concentration $c = 2.90\ \mathrm{mg\,mL^{-1}}$). (a) Sedimentation coefficient distribution obtained with SV-AUC for the BSA solution (76.9 % of monomer, 18.3 % of dimer, 3.7 % of trimer, and 1.1 % of tetramer). (b) Guinier plot for the BSA. Open circles are the experimental SAXS intensity and a solid line represents Guinier

fitting within 0.0008 Å^{-2} ≤ q^2 ≤ 0.0015 Å^{-2}, resulting in $R_{\mathrm{ge}} = 33.4 \pm 0.3$ Å (cf. $R_{\mathrm{g}} = 27.1$ Å from the crystal structure) and $i_{\mathrm{exp}}(0) = 0.0630 \pm 0.0005\ \mathrm{mg^{-1}cm^2}$ (cf. $I(0) = 0.0465\ \mathrm{mg^{-1}cm^2}$ from the crystal structure). (c) Residuals between experimental data and Guinier fitting line; $(I_{\mathrm{exp}}(q) - I_{\mathrm{fit}}(q))/I_{\mathrm{exp}}(q)$, where $I_{\mathrm{exp}}(q)$ and $I_{\mathrm{fit}}(q)$ are the experimental data and the Guinier fitting, respectively. The deviation of experimental data form Guinier approximation was increased toward the lower q range.

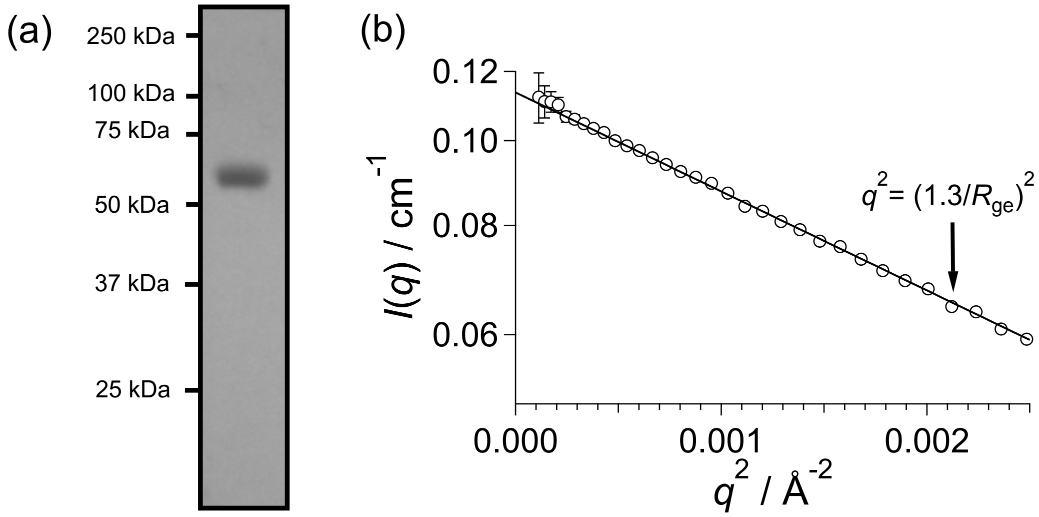

Supplementary Fig.2. Sample quality for this study. (a) SDS-PAGE and (b) Guinier plots of SAXS profile of a purified BSA solution (BSA1), which was used for this study (see METHODS). (a) In the SDS-PAGE, the BSA was detected as single band by Coomassie Brilliant Blue staining. It looks that no clear aggregation and contamination was observed. (b) The scattering profile follows a fine straight line in the Guinier plot and a black line shows the result of the least square fitting with Guinier formula: The obtained R_{ge}, $i_{\mathrm{exp}}(0)$, and the molecular weight M are listed in Supplementary Table 1.

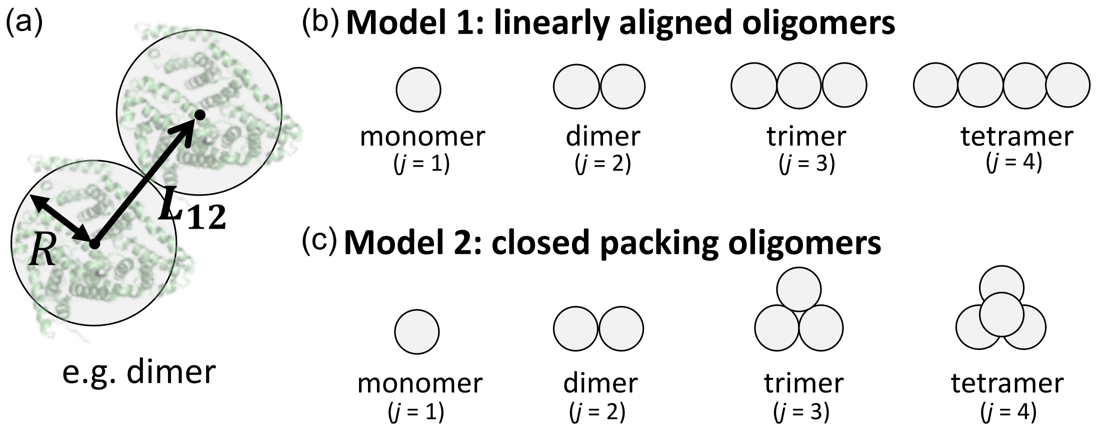

Supplementary Fig.3. Linearly aligned oligomers (Model 1) and closed packing oligomers (Model 2). (a) Monomer configurations. Monomer has an average residential radius R. The vectors between the centers of gravity of l-th and m-th monomers are defined as $\boldsymbol{L}_{lm}$ (see Supplementary Note 2-1). (b) Oligomers in Model 1 and (c) oligomers in Model 2.

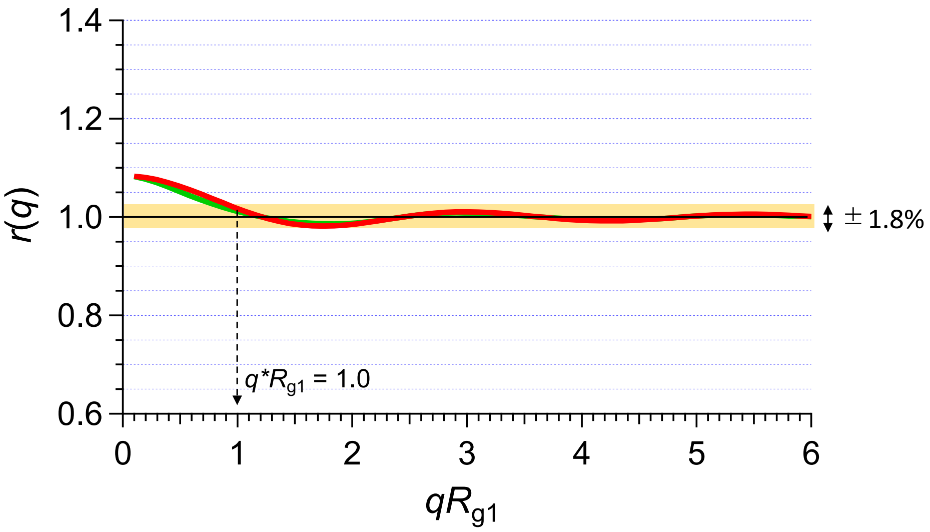

Supplementary Fig.4. Scattering intensity ratios $r(q)$ (eqs.(S10) and (S13) in Supplementary Note 2-1) based on the oligomer models: a green and red lines correspond to $r(q)$ for linearly aligned model (Model 1) and closed packing model (Model 2)) calculated with the $\{c_j\}$ of the demonstrated BSA1, respectively.

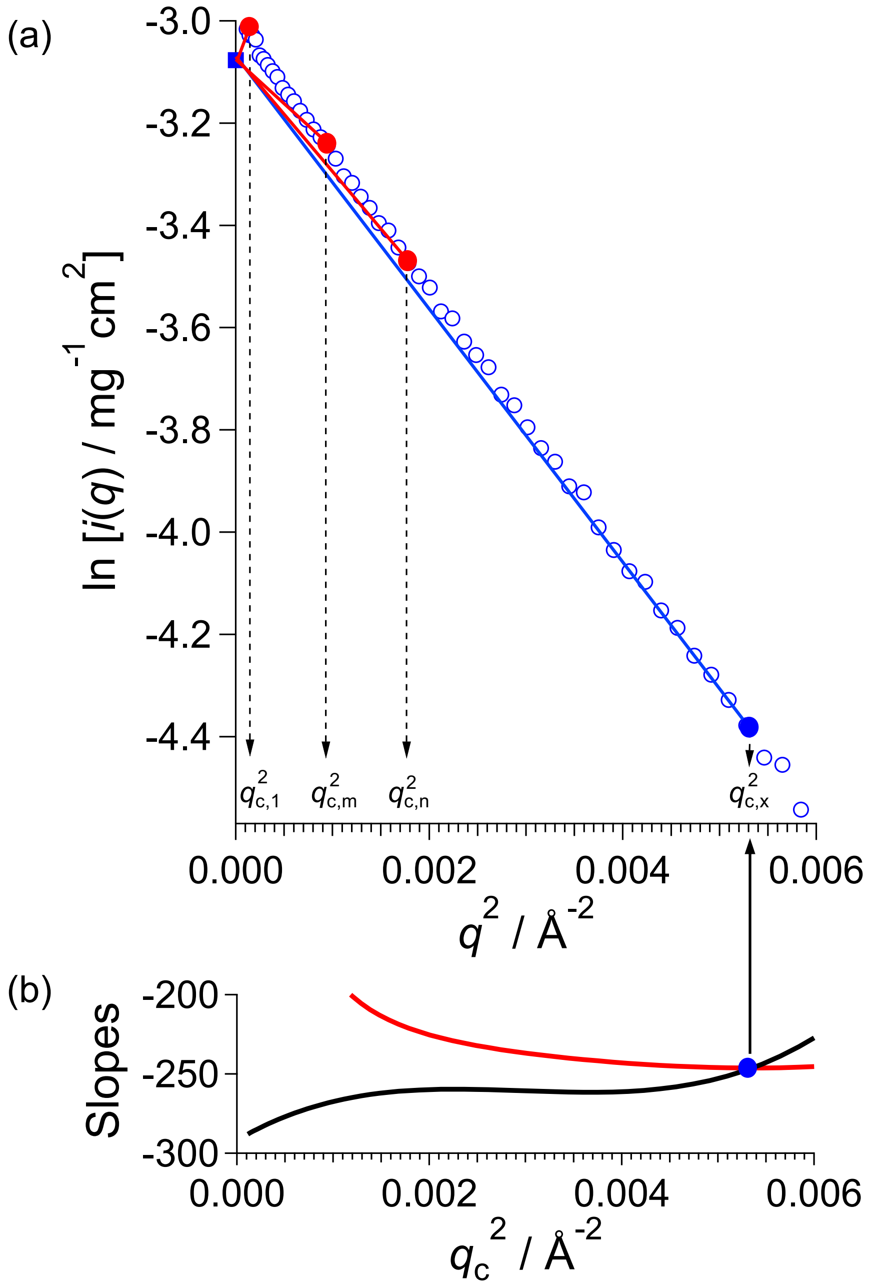

Supplementary Fig.5. (a) Open blue circles denote the extrapolated $i_{1\mathrm{h}}(q_\mathrm{l})^*$, red lines show the candidates of Guinier plots, $i_{1\mathrm{l}}(q_\mathrm{l})^*$, connecting $i_{1\mathrm{h}}\left(q_{\mathrm{c},j}\right)^*$ to $i_1(0)$ obtained in Step 3 (closed blue square), and a blue line does the smooth connected $i_{1\mathrm{l}}(q_\mathrm{l})^*$ for the demonstrated BSA1, respectively. Here, the closed red circles are the candidates of connection points $q_{c,j}^2$: the closed blue circle is the smooth connection point $q_{c,x}^2$. (b) Slopes of $\ln(i_{1\mathrm{l}}(q_\mathrm{l})^*)$ (red line) and the extrapolated $\ln(i_{1\mathrm{h}}(q_\mathrm{l})^*)$ (black line) as a function of q_c^2 (see Supplementary Note 3-1).

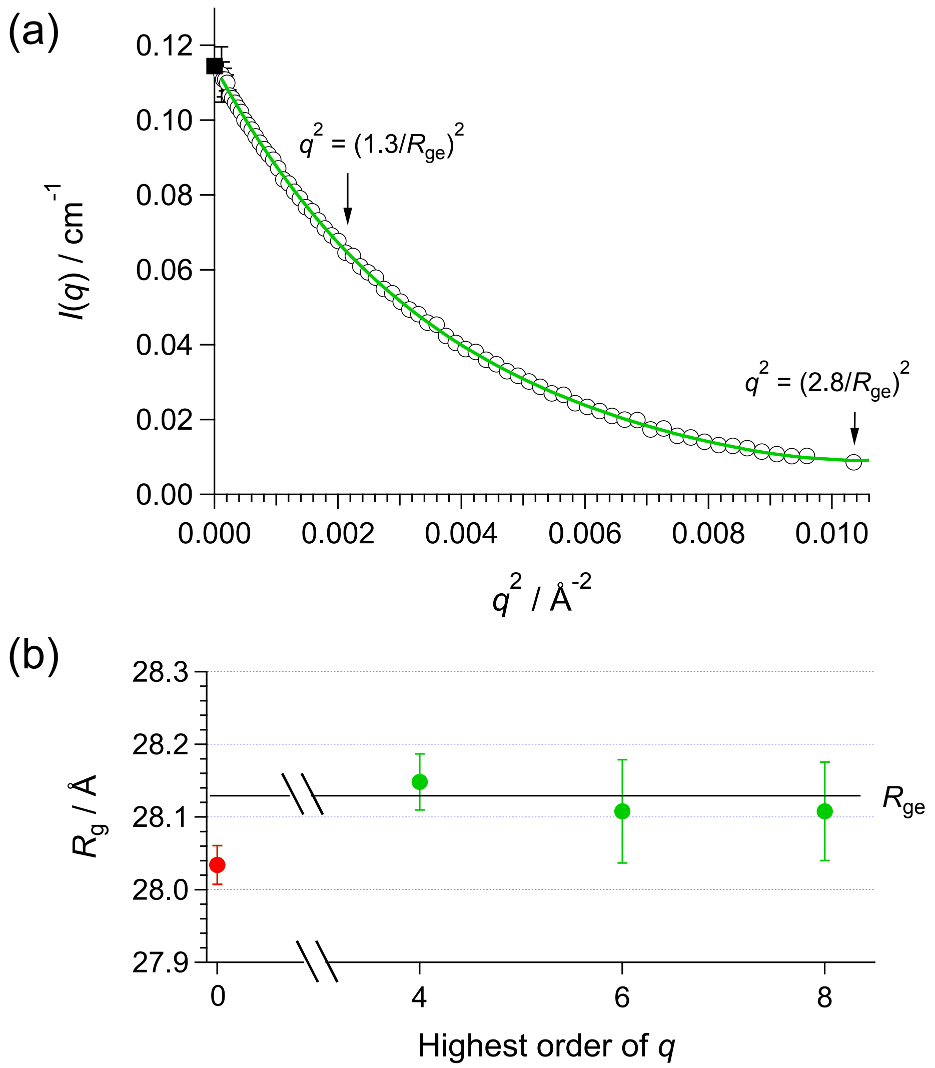

Supplementary Fig.6. Expanded Guinier formula. (a) Scattering intensity $ci_{\text{exp}}(q)$ (black circles) and the least square fitting of the expanded Guinier formula with q^8-order of $D_{\text{c}}(q)$ (green curve) for BSA1. (b) Obtained R_{g} as a function of q^2-order of $D_{\text{c}}(q)$. Red and green circles are the least square fitting results of simple and expanded Guinier formula, respectively. The solid line shows R_{ge} obtained from the Guinier analysis in the standard Guinier range $q < 1.3/R_{\text{ge}}$ (see Supplementary Note 3-2 and Supplementary Table 1).

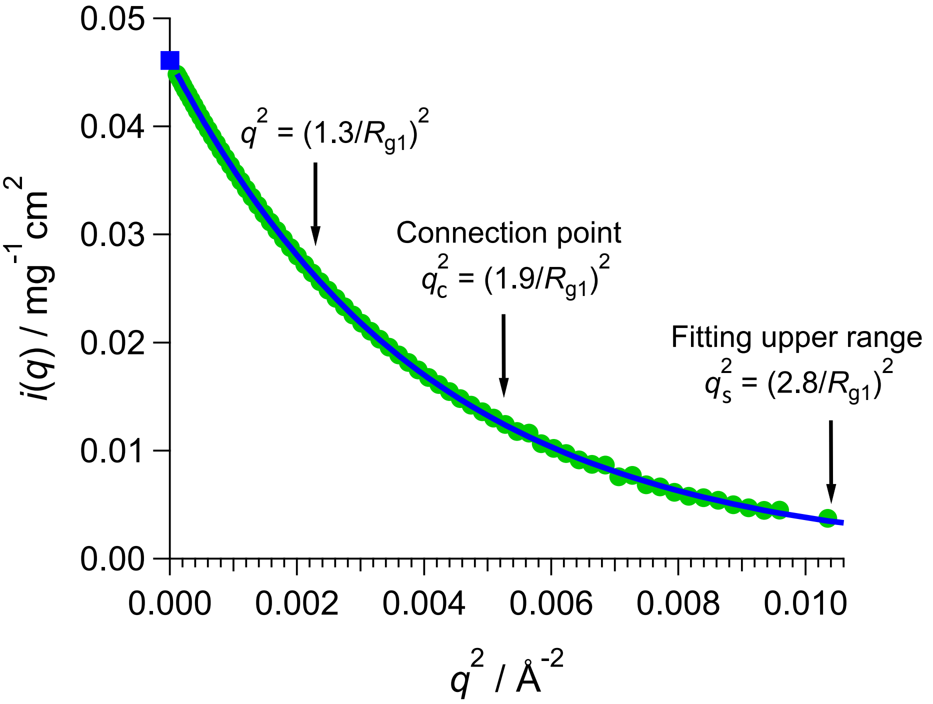

Supplementary Fig.7. Refinement of initial intensity with the expanded Guinier formula. Green circles and blue square show the initial intensity $i_1(q)^*$ and the forward scattering $i_1(0)$ for BSA1 sample. A blue line expresses the result of the least square fitting with the expanded Guinier formula with q^8-ordered correction term $D_c(q)$ in $q^2 \leq (2.8/R_{g1})^2$ see Supplementary Note 3-3).

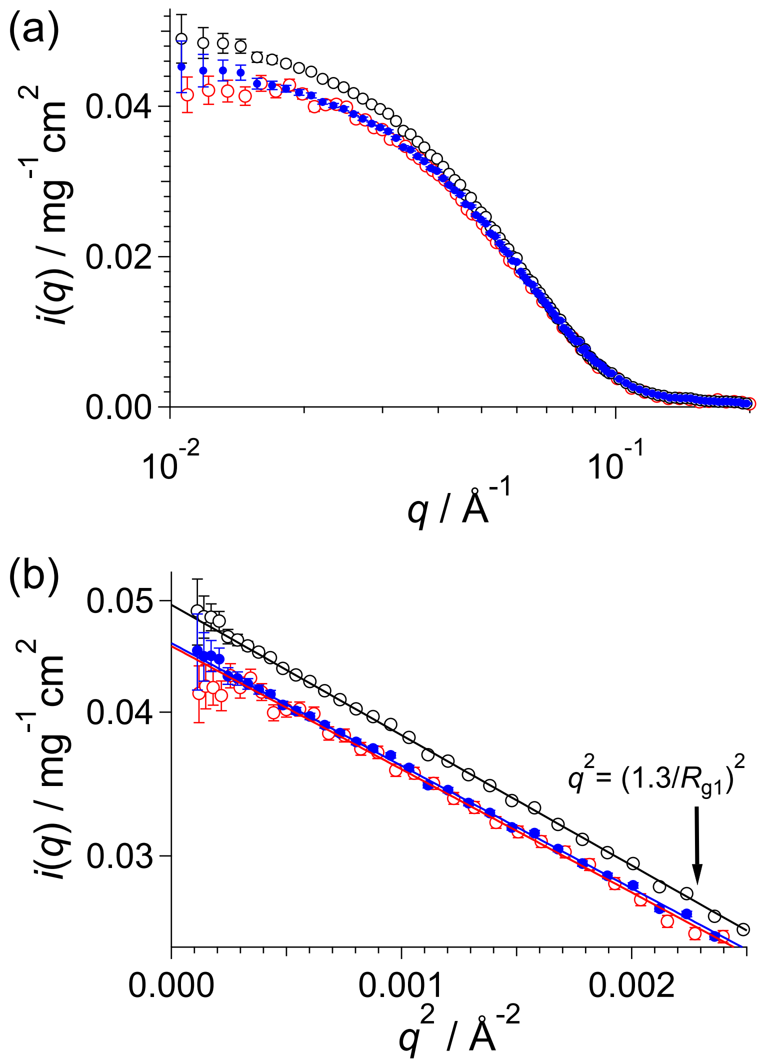

Supplementary Fig.8. Comparison between non-treated SAXS, AUC-SAXS, and SEC-SAXS. (a) SAXS intensities and (b) their Guinier plots for BSA1: open black, closed blue, and open red circles show non-treated SAXS $i_{\mathrm{exp}}(q)$, the extracted monomer SAXS by AUC-SAS $i_1(q)$, and SEC-SAXS[1], respectively. The straight lines in panel (b) represent the least square fitting with Guinier formula. The gyration radii and the forward scattering intensities are listed in Supplementary Table 1.

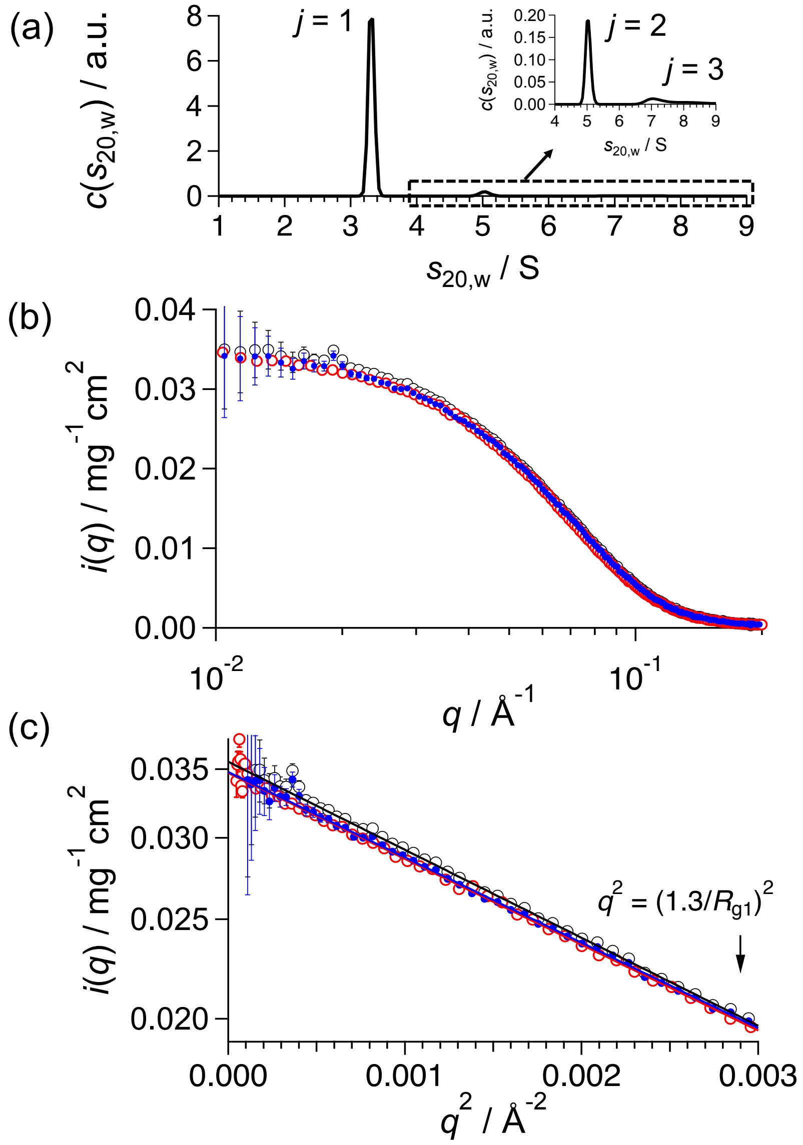

Supplementary Fig.9. AUC-SAS for ovalbumin (OVA) solution. The OVA solution sample contain small amounts of aggregates: The purification procedures are described in METHODS. (a) Sedimentation coefficient distribution obtained with SV-AUC for the OVA solution. (b) SAXS intensities for the OVA solution. Open black circles, open red circles, and closed blue circles represent the intensities with non-treated SAXS, SEC-SAXS[1], and AUC-SAS, respectively. (c) Their Guinier plots. As listed in Supplementary Table 1, the experimentally obtained R_{ge} and $i_{exp}(0)$ were larger than those by SEC-SAXS[1] due to the effect of aggregation, while R_{g1} and $i_1(0)$ after AUC-SAS-treatment well agree with them.

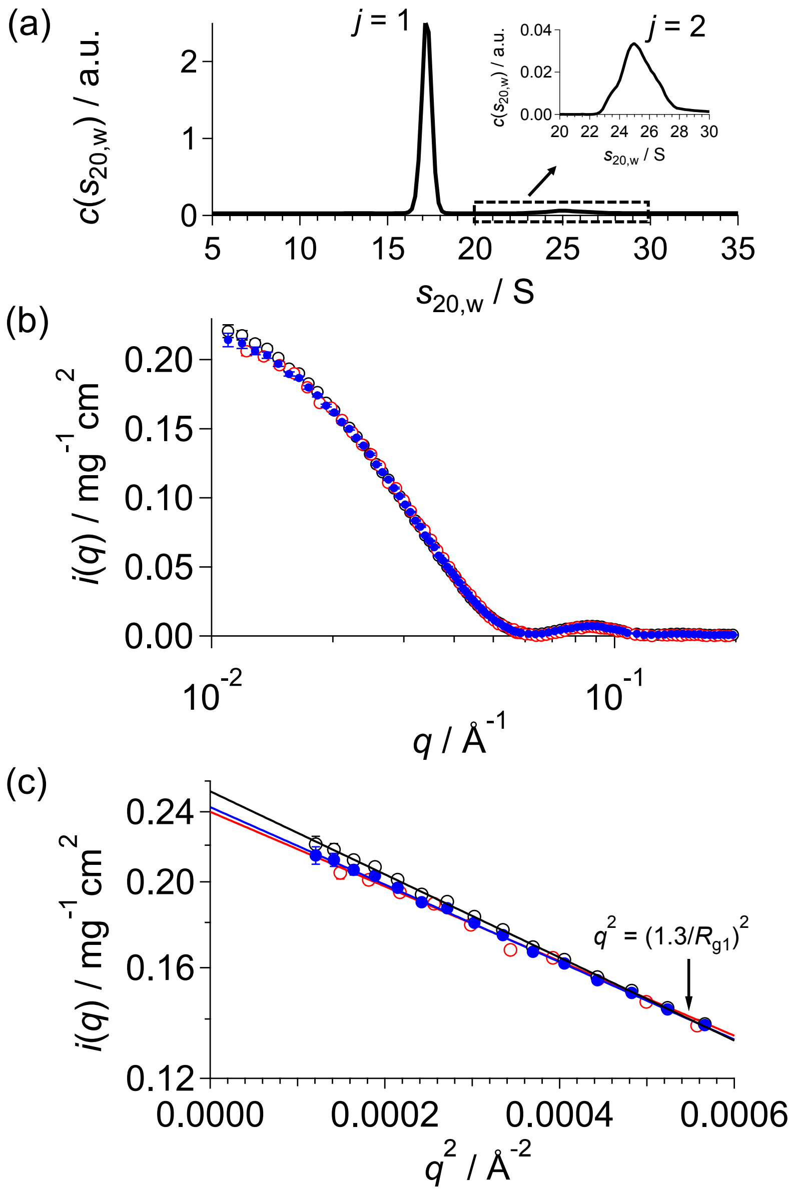

Supplementary Fig.10. AUC-SAS for apoferritin (AF) solution. The AF solution sample contain small amounts of aggregates: The purification procedures are described in METHODS. (a) Sedimentation coefficient distributions obtained with SV-AUC for the AF solution. (b) SAXS intensities for the AF solution. Open black circles, open red circles, and closed blue circles represent the intensities with non-treated SAXS, SEC-SAXS[1], and AUC-SAS, respectively. (c) Their Guinier plots. As listed in Supplementary Table 1, the experimentally obtained R_{ge} and $i_{\mathrm{exp}}(0)$ were larger than those by SEC-SAXS[1] due to the effect of aggregation, while R_{g1} and $i_1(0)$ after AUC-SAS-treatment well agree with them.

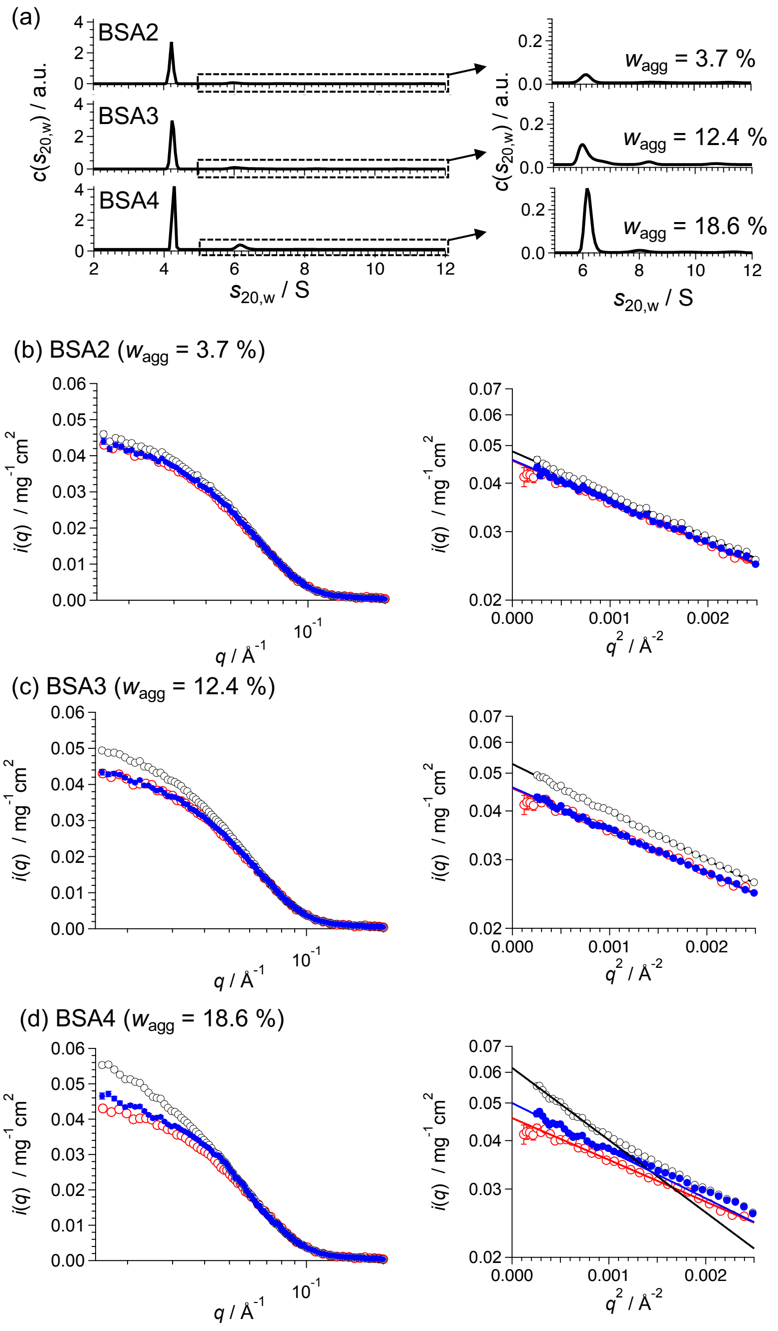

Supplementary Fig.11. (a) Sedimentation coefficient distributions obtained from SV-AUC for the BSA containing various amounts of aggregates. (b)-(d) SAXS intensities and their Guinier plots for BSA2-4, respectively. Open black, open red, and closed blue circles represent the intensities with non-treated SAXS, SEC-SAXS[1], and AUC-SAS, respectively. Gyration radii and forward scattering intensities obtained with Guinier analysis are listed in Supplementary Table 3. Applicable boundary is located between 12.4 % and 18.6 % (see Supplementary Note 4-1).

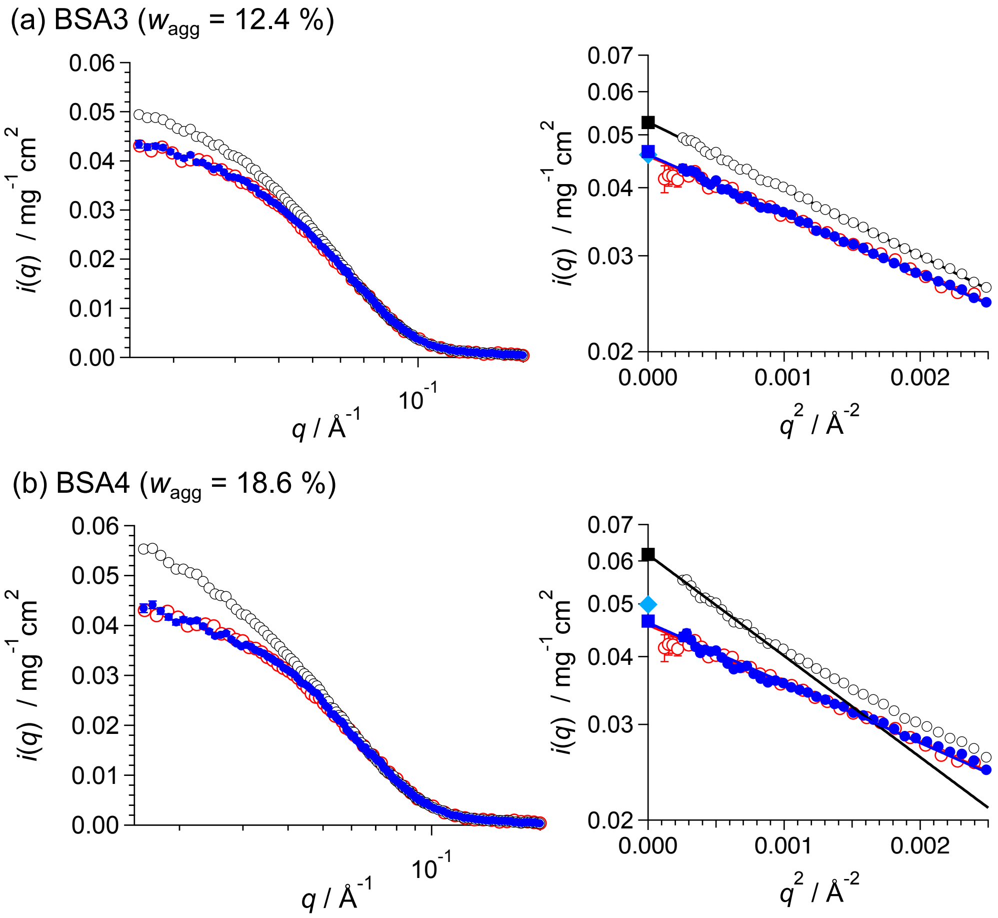

Supplementary Fig.12. Possible improvement for higher concentration of aggregates (see Supplementary Note 4-2). SAXS intensities (left) and their Guinier plots (right) for the (a) BSA3 and (b) BSA4, respectively. Open black circles, open red circles, and closed blue circles represent the intensities with non-treated SAXS, SEC-SAXS[1], and improved AUC-SAS, respectively. Closed black square, closed cyan diamond, and closed blue square express $i_{\mathrm{exp}}(0)$, $i_1(0)$ obtained with the present AUC-SAS protocol, $t_1 c i_{\mathrm{exp}}(0)$, and $i_1(0)$ calculated by the improved approach with eq.(S21), respectively. Gyration radii and forward scattering intensities obtained with the Guinier analysis are listed in Supplementary Table 3.

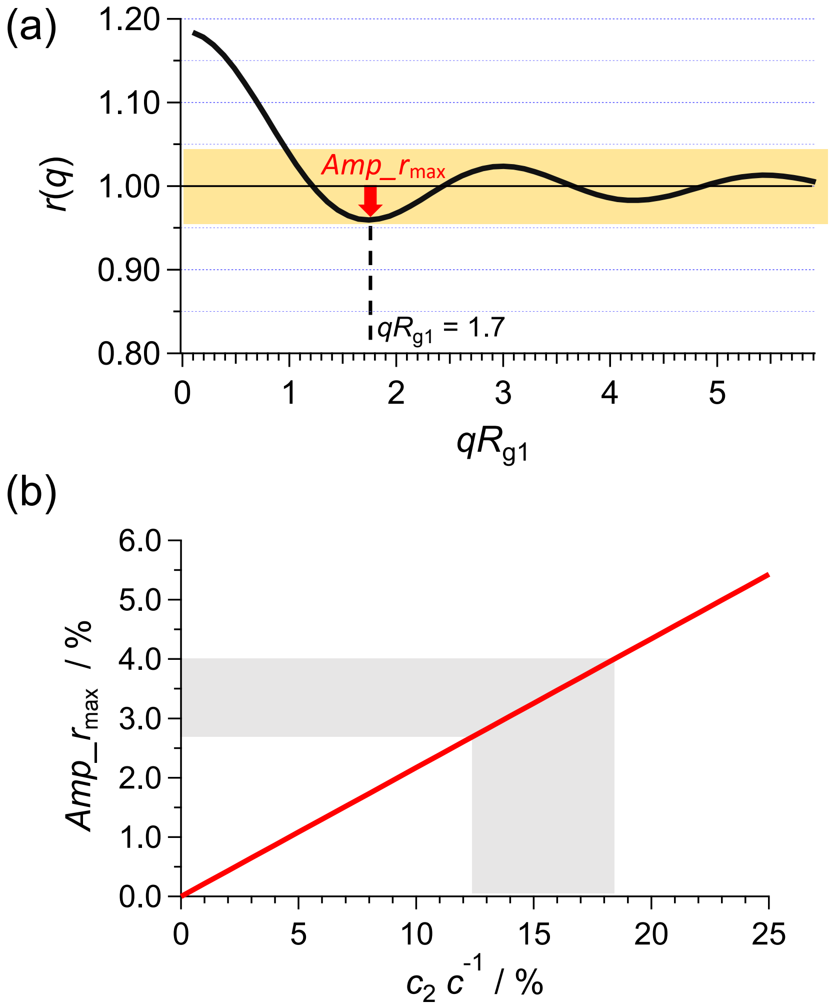

Supplementary Fig.13. (a) Scattering intensity ratio $r(q)$ calculated with eq.(S10) with $c_2/c = 0.186$ and $c_3 = c_4 = 0$. (b) The aggregates amount dependence of the $Amp\_r_{\max}$ (see Supplementary Note 4-3).

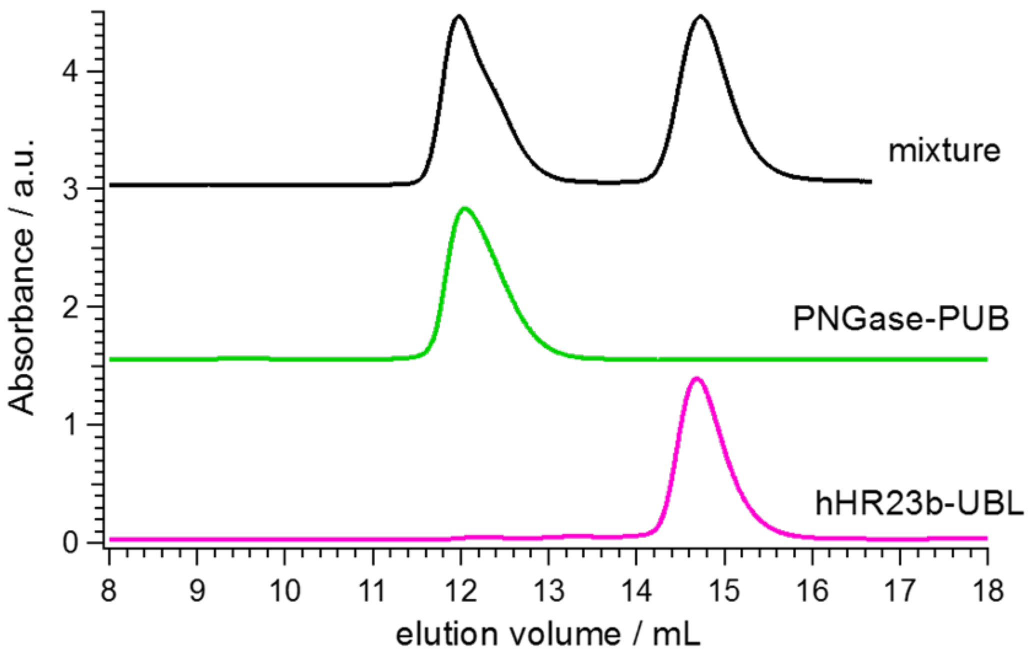

Supplementary Fig.14. SEC charts for hHR23b-UBL (pink), PNGase-PUB (green), and the mixture (black) at 25 ºC. The peak from the complex was not observed in the mixture, meaning that SEC broke this weakly-bound complex out. The SEC experiments for hHR23b-UBL, PNGase-PUB and their mixture were performed with Prominence HPLC system (SHIMADZU) with 10 mM sodium phosphate buffer (pH 7.0). Superdex 75 increase 10/300GL (GE Healthcare) was used for a separation column. The flow rate was set at 0.75 mL/min. The all sample solutions were injected at 500 μL volume. The concentrations of the injected solutions were 0.50 mg/mL for hHR23b-UBL, 0.30 mg/mL for PNGase-PUB, and [hHR23b-UBL: 0.95 mg/mL (100 μM) + PNGase-PUB: 1.34 mg/mL (100 μM)] for the mixture.

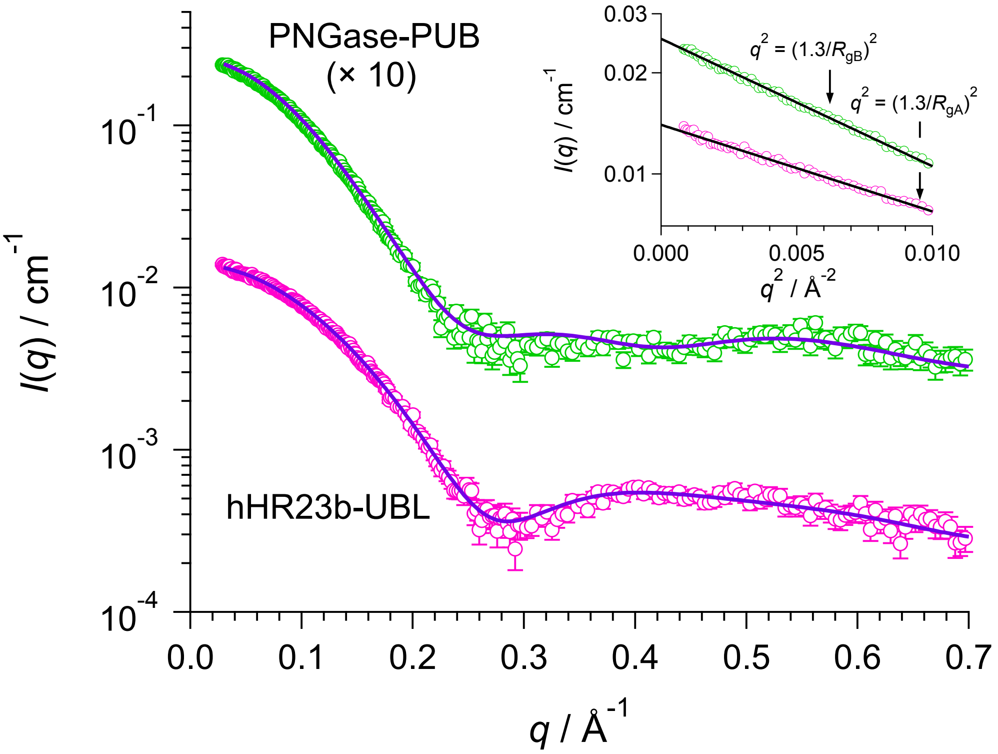

Supplementary Fig.15. SAXS intensities for hHR23b-UBL (pink) at 1.90 mg/mL (200 μM) and PNGase-PUB (green) at 2.68 mg/mL (200 μM) in 10 mM sodium phosphate buffer (pH 7.0). The purple curves show the SAXS intensities calculated from their structures determined by NMR[2] (PDB code 1P1A for hHR23b-UBL and 2D5U for PNGase-PUB). The intensity for PNGase-PUB is ten times shifted along to the vertical axis. The inset displays the Guinier plots and the Guinier fitting lines. Gyration radii and forward scattering intensities were listed in Supplementary Table 4.

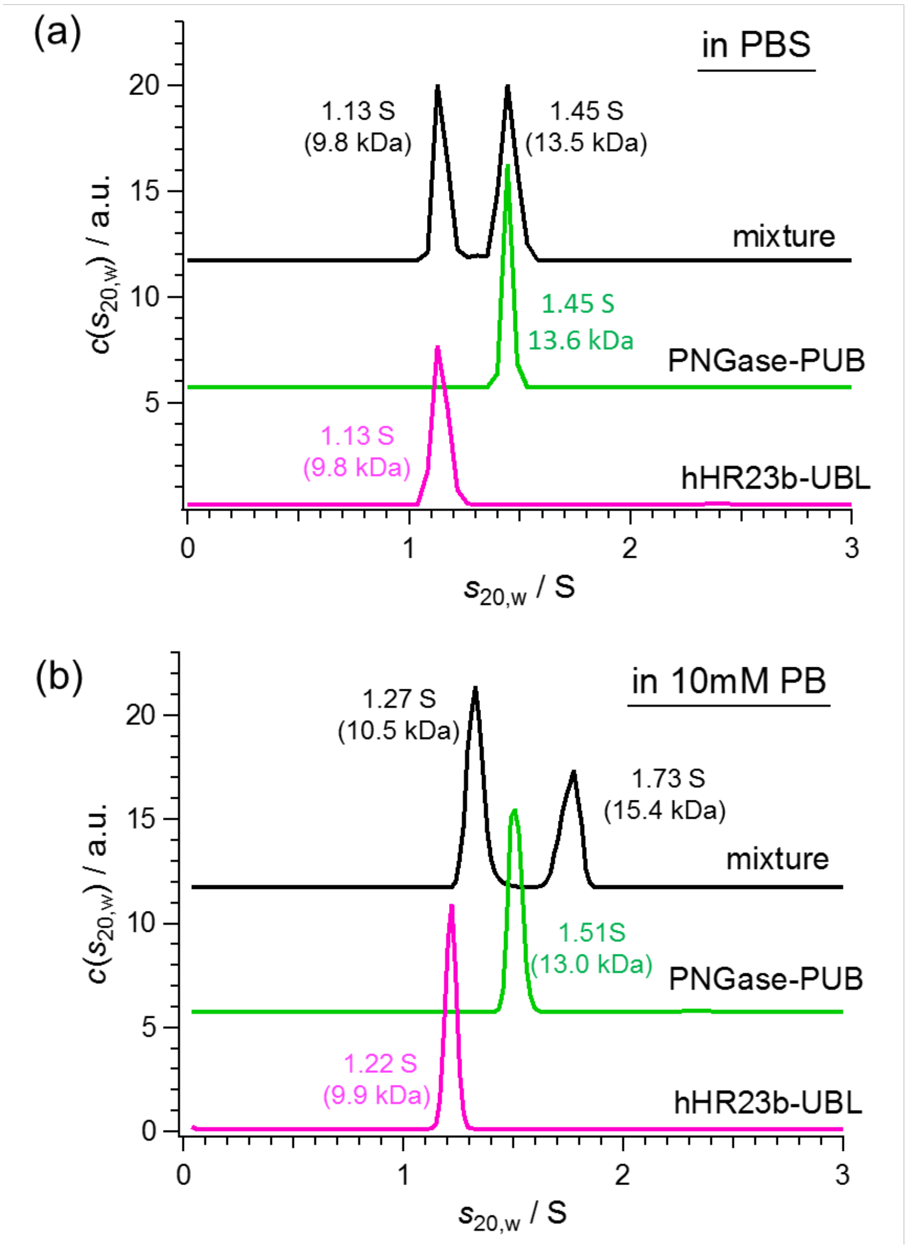

Supplementary Fig.16. Sedimentation coefficient distributions obtained with SV-AUC for hHR23b-UBL at 1.90 mg/mL (200µM) (pink), PNGase-PUB at 2.68 mg/mL (200µM) (green) and their mixture with hHR23b-UBL (1.90 mg/mL, 200 µM) and PNGase-PUB (2.68 mg/mL, 200 µM) (black) (a) in PBS (pH 7.4) and (b) in 10 mM PB (pH7.0).

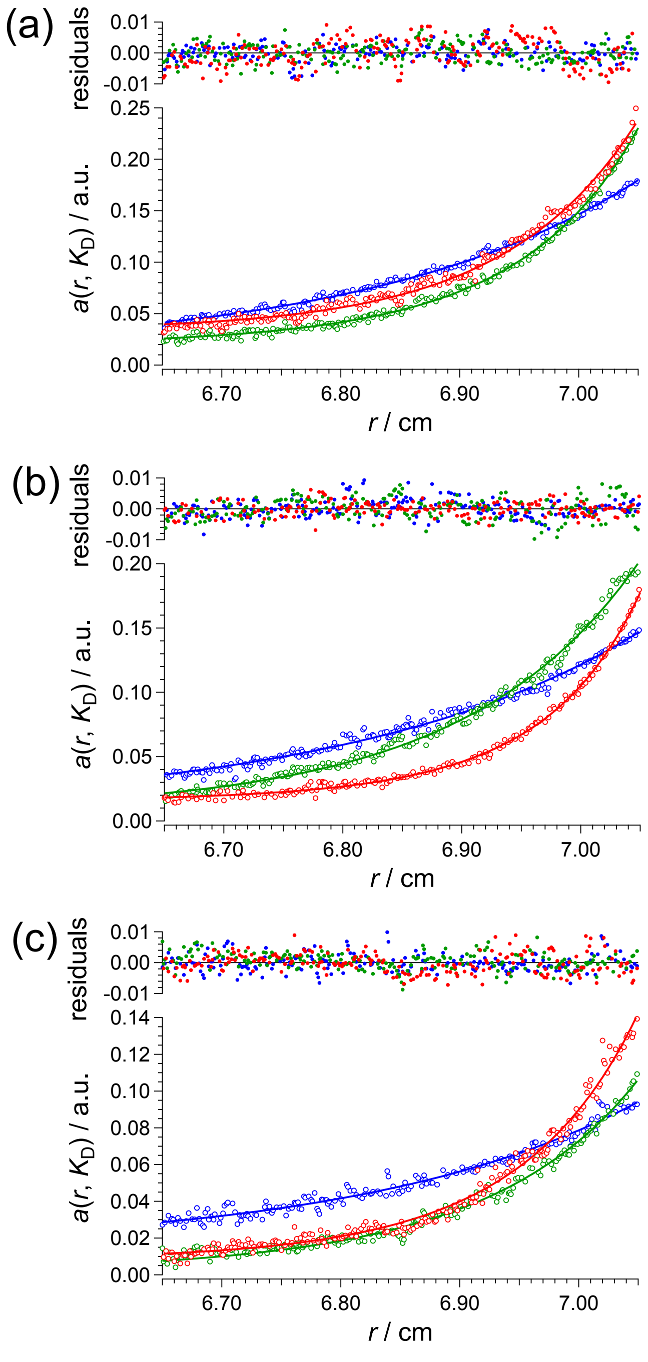

Supplementary Fig.17. Absorbance gradient at sedimentation equilibrium given by SE-AUC. (a) [hHR23b-UBL] = [PNGase-PUB] = 100μM, (b) [hHR23b-UBL] = [PNGase-PUB] = 75μM, and (c) [hHR23b-UBL] = [PNGase-PUB] = 50μM. Blue, green, and red open circles show the result at 20000, 30000, and 35000 rpm, respectively. Solid lines represent the fitting curves with eq.(7). Upper panel in each graph shows the residuals between experimental data and fitting curves.

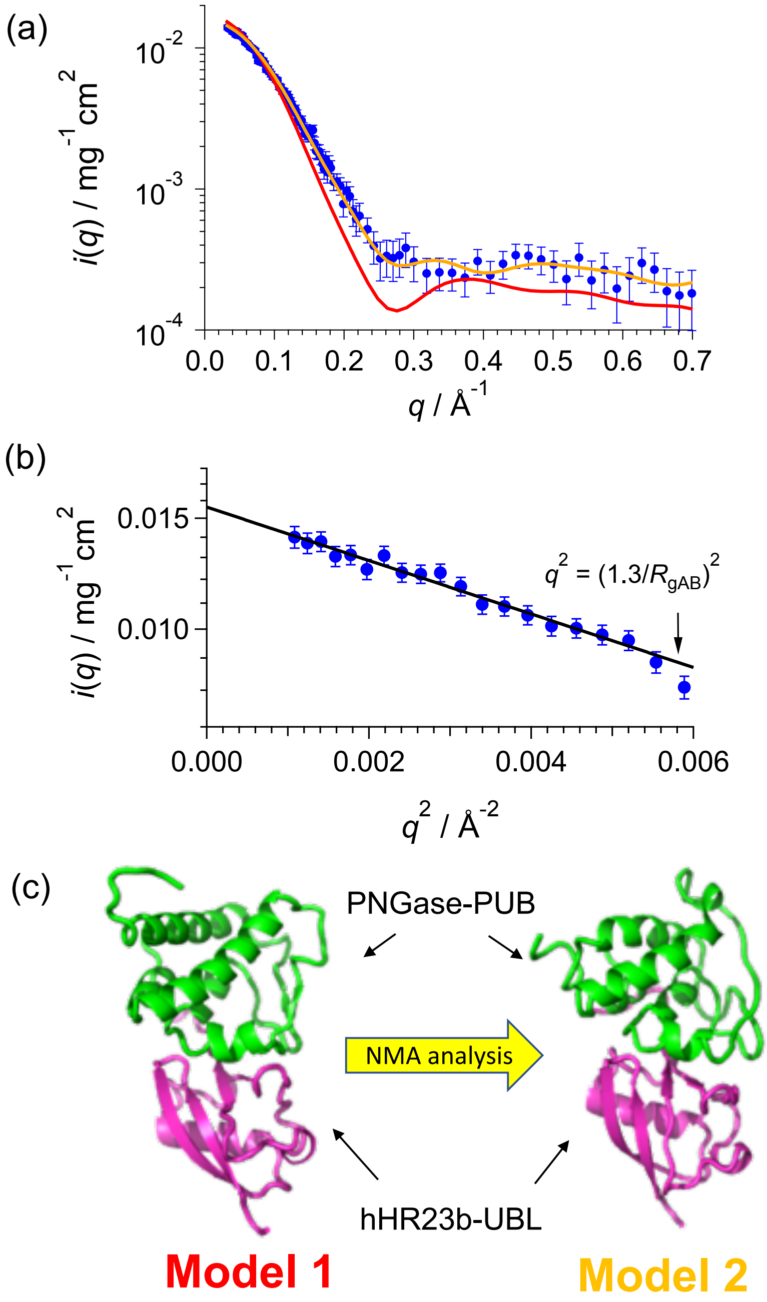

Supplementary Fig.18. (a) AUC-SAS-treated scattering intensity $i_{AB}(q)$ (blue circles) for the complex of hHR23b-UBL and PNGase-PUB. The red and orange lines represent the scattering intensities calculated from Models 1 and 2, respectively (see panel (c)). (b) Guinier plot. Solid line expresses the least square fitting with Guinier approximation. (c) Structure models of complex. Model 1 is the initial model based on NMR result[2] and Model 2 is its refined structure by utilizing NMA analysis[3,4].

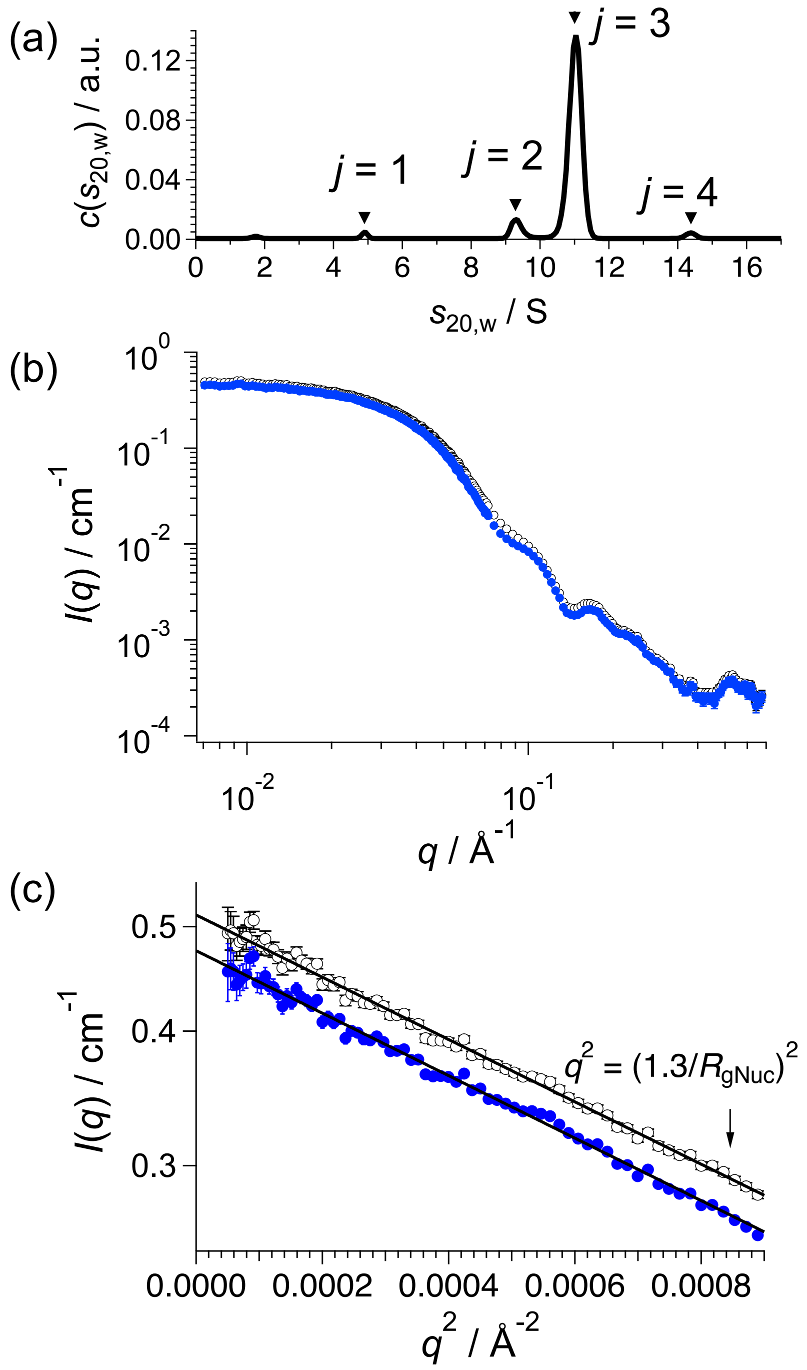

Supplementary Fig.19. (a) Sedimentation coefficient distribution obtained from SV-AUC for the nucleosome solution. (b) SAXS intensities and (c) the Guinier plots for the nucleosome solution. Open black and closed blue circles represent the non-treated SAXS intensity $ci_{\mathrm{exp}}(q)$ and the extracted SAXS intensity $c_{\mathrm{Nuc}}i_{\mathrm{Nuc}}(q)$ with AUC-SAS, respectively. Gyration radii and forward scattering intensities are listed in Supplementary Table 7.

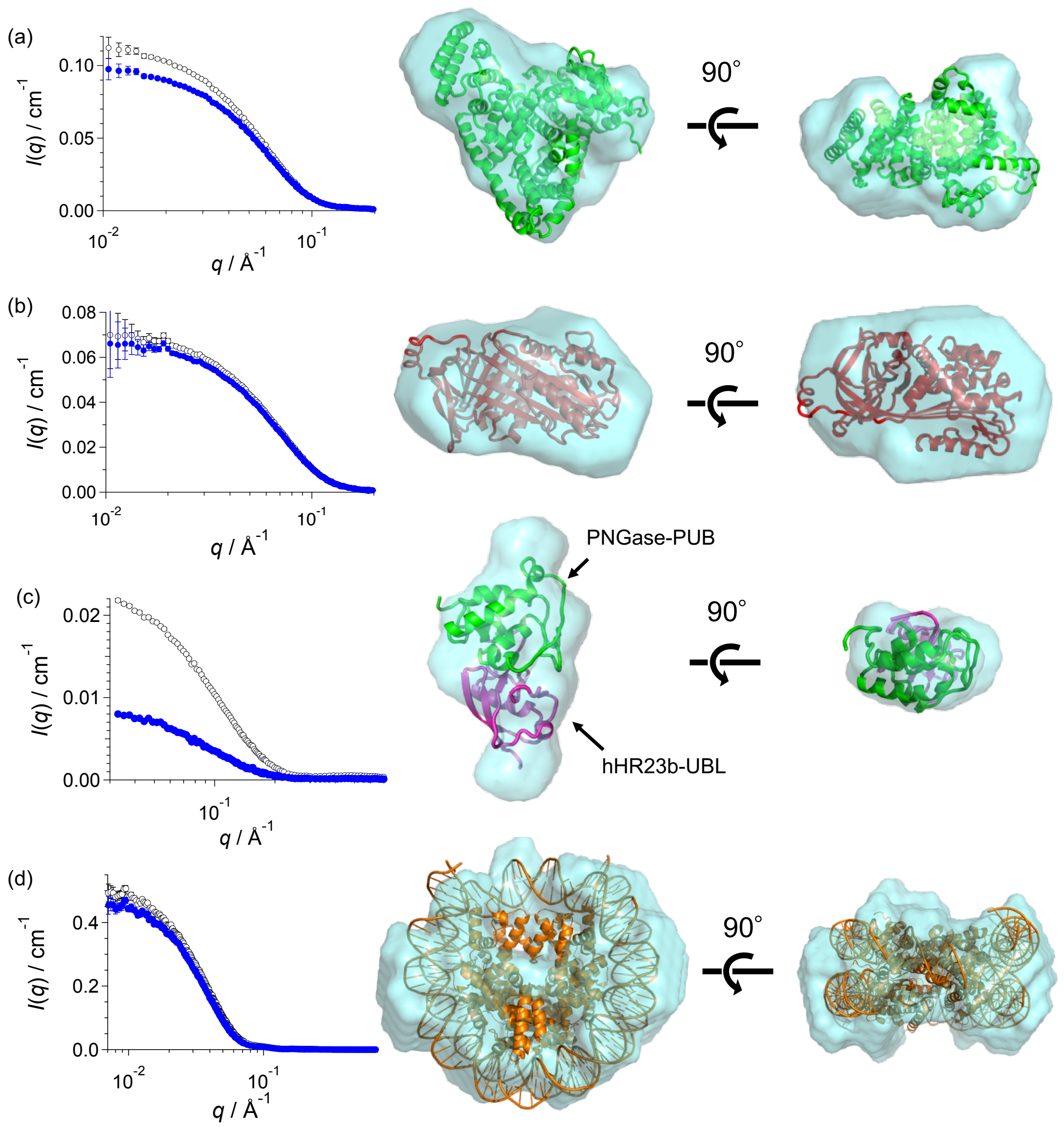

Supplementary Fig.20. Scattering profile with AUC-SAS and *ab initio* models obtained from them. The *ab initio* models were calculated with DAMMIN software[5] via calculating the distance distribution function $P(r)$ with GNOM software[6]. (L) Scattering intensities and (R) their *ab initio* models of (a) BSA, (b) OVA, (c) hHR23b-UBL + PNGase-PUB complex, and (d) the nucleosome. (L) Open black and closed blue circles show the scattering intensities of non-treated SAXS and AUC-SAS, respectively. (R) The *ab initio* models are calculated with AUC-SAS intensities. The crystal structures for (a) BSA; 4F5S[7], (b) OVA; 1OVA[8], and (d) the nucleosome; 3AFA[9] and (c) the refined structure with normal mode analysis are superposed on the *ab initio* models.

SUPPLEMENTARY TABLES

Supplementary Table 1. Structural parameters of BSA1, OVA, and AF obtained by non-treated SAXS, AUC-SAS, SEC-SAXS and crystal structure.

BSA1 (demonstrated in the main text)

	R_{gx} / Å	$i_x(0)$ / $10^{-2}\mathrm{mg}^{-1}\mathrm{cm}^2$	M / kDa	χ^2
Non-treated SAXS	28.1 ± 0.2	5.00 ± 0.01	74.5 ± 0.1	1.01
AUC-SAS	27.2 ± 0.2	4.61 ± 0.01	68.7 ± 0.1	0.98
SEC-SAXS[1]	27.3 ± 0.3	4.50 ± 0.04	67.1 ± 0.1	1.25
Crystal (4F5S)[7]	27.1	4.65	69.2	-

OVA

	R_{gx} / Å	$i_x(0)$ / $10^{-2}\mathrm{mg}^{-1}\mathrm{cm}^2$	M / kDa	χ^2
Non-treated SAXS	24.4 ± 0.2	3.59 ± 0.01	49.3 ± 0.1	1.07
AUC-SAS	23.9 ± 0.2	3.46 ± 0.01	47.4 ± 0.1	1.04
SEC-SAXS[1]	23.9 ± 0.4	3.44 ± 0.04	47.2 ± 0.4	1.47
Crystal (1OVA[8])	22.8	3.14	43.0	-

AF

	R_{gx} / Å	$i_x(0)$ / $10^{-1}\mathrm{mg}^{-1}\mathrm{cm}^2$	M / kDa	χ^2
Non-treated SAXS	56.7 ± 0.4	2.53 ± 0.01	412 ± 2	0.82
AUC-SAS	54.5 ± 0.5	2.43 ± 0.01	396 ± 2	0.81
SEC-SAXS[1]	53.9 ± 0.9	2.42 ± 0.03	394 ± 4	0.98
Crystal (4V1W[10])	54.0	2.93	477	-

R_{gx}: gyration radii (R_{ge}: non-treated SAXS, R_{g1}: monomer), $i_x(0)$: forward scattering intensities normalized by mass concentration ($i_{exp}(0)$: non-treated SAXS, $i_1(0)$: monomer), M : molecular weight calculated with $i_x(0)$[11]. χ^2: chi-squared value for the least square fitting with Guinier approximation.

Supplementary Table 2. Distribution of oligomers in solution.

BSA1 (demonstrated in the main text)

j	$s_{20,\mathrm{w},j}$ / S	M_j / kDa	w_j / %	c_j / mg mL^{-1}	t_j
1	4.3	69.4	94.1	2.154	0.868
2	6.8	140	4.2	0.097	0.078
3	9.0	209	0.9	0.021	0.025
4	10.1	276	0.8	0.018	0.029

OVA

j	$s_{20,\mathrm{w},j}$ / S	M_j / kDa	w_j / %	c_j / mg mL^{-1}	t_j
1	3.3	46.0	96.5	1.930	0.931
2	5.0	86.7	3.3	0.066	0.064
3	7.1	140	0.2	0.004	0.005

AF

j	$s_{20,\mathrm{w},j}$ / S	M_j / kDa	w_j / %	c_j / mg mL^{-1}	t_j
1	17.1	487	95.0	1.720	0.914
2	25.0	871	5.0	0.090	0.086

j: aggregation-degree, $s_{20,\mathrm{w},j}$: sedimentation coefficient, M_j: molecular weight, w_j: weight fraction, c_j: mass concentration, t_j: contribution ratio in the forward scattering to the whole forward scattering intensity. The concentrations of BSA, OVA, and AF were $c = 2.29\ \mathrm{mg\ mL^{-1}}$, $2.00\ \mathrm{mg\ mL^{-1}}$, $1.81\ \mathrm{mg\ mL^{-1}}$. The frictional ratios f_r for BSA, OVA, and AF were 1.38, 1.33, and 1.25, respectively.

Supplementary Table 3. Structural parameters of BSA2-4 obtained by non-treated SAXS, AUC-SAXS, and improved AUC-SAS.

BSA2-4 (demonstrated in Supplementary Note 4)

	Sample	w_{agg} / %	$R_{\mathrm{g}x}$ / Å	$i_x(0)$ / $10^{-2}\mathrm{mg}^{-1}\mathrm{cm}^2$	M / kDa	χ^2
Non-treated SAXS	BSA2	3.7	27.6 ± 0.1	4.83 ± 0.01	72.0 ± 0.1	0.99
	BSA3	12.4	29.1 ± 0.1	5.27 ± 0.01	78.5 ± 0.1	1.00
	BSA4	18.6	35.9 ± 0.5	6.17 ± 0.03	91.9 ± 0.3	1.51
AUC-SAS	BSA2	3.7	27.2 ± 0.1	4.60 ± 0.01	68.6 ± 0.1	0.98
	BSA3	12.4	27.4 ± 0.1	4.60 ± 0.01	68.6 ± 0.1	1.00
	BSA4	18.6	29.2 ± 0.5	4.99 ± 0.03	74.4 ± 0.3	1.15
Improved AUC-SAS	BSA2	3.7	27.2 ± 0.1	4.65 ± 0.01	69.2 ± 0.1	0.98
	BSA3	12.4	27.4 ± 0.1	4.65 ± 0.01	69.2 ± 0.1	1.00
	BSA4	18.6	27.6 ± 0.3	4.65 ± 0.01	69.2 ± 0.1	0.96

w_{agg}: weight fraction of aggregation (dimer + trimer + tetramer). $R_{\mathrm{g}x}$: gyration radii (R_{ge}: non-treated SAXS, R_{g1}: others), $i_x(0)$: forward scattering intensities normalized by mass concentration ($i_{\mathrm{exp}}(0)$: non-treated SAXS, $i_1(0)$: others), M : molecular weight calculated with $i_x(0)$[11]. χ^2: chi-squared value for the least square fitting with Guinier approximation.

Supplementary Table 4. Concentrations and structural parameters of hHR23b-UBL, PNGase-PUB, their mixture, and the complex in the mixture.

	Concentration	R_{gx} / Å	$i_x(0)$ / $10^{-3}\mathrm{mg}^{-1}\mathrm{cm}^2$	M_x / kDa	χ^2
hHR23b-UBL	1.90 mg/mL (200 μM)	13.2±0.1	7.32±0.05	9.7±0.1	1.00
PNGase-PUB	2.68 mg/mL (200 μM)	16.2±0.1	9.44±0.03	12.5±0.1	1.07
Mixture	0.95 mg/mL + 1.34 mg/mL (100μM + 100μM)	16.0±0.1	8.84±0.03	11.7±0.1	1.06
Complex	0.57 mg/mL (25 μM)	17.1±0.4	15.7±0.4	20.8±0.6	0.86

Concentration and structural parameters of hHR23b-UBL, PNGase-PUB and their mixture were individually measured (see Fig.6(a) and Supplementary Fig.15). Concentration of the complex was calculated with $K_\mathrm{D} = 227 \pm 28$ μM obtained with SE-AUC and the structure parameters of the complex was obtained with AUC-SAS. R_{gx}: gyration radii. $i_x(0)$: forward scattering intensities normalized by mass concentration. M_x: molecular weights calculated with $i_x(0)$[11] ($x = \mathrm{A, B, exp, AB}$: experimental value for hHR23b-UBL, PNGase-PUB, Mixture, and complex obtained with AUC-SAS, respectively). χ^2 : chi-squared value for the least square fitting with Guinier approximation.

Supplementary Table 5. Chi-squared value χ^2 for the least square fitting to SE-SUV data with eq.(7) (see Supplementary Fig.17 and Supplementary Note 6).

Concentration	20000 rpm	30000 rpm	35000 rpm
100μM + 100μM	3.77	3.85	4.38
75 μM + 75 μM	4.58	5.21	3.58
50 μM + 50 μM	6.11	5.12	5.93

Supplementary Table 6. Distribution of components in the nucleosome solution.

j	$s_{20,\mathrm{w},j}$ / S	M_j / kDa	w_j / %	c_j / mg mL^{-1}	t_j
1 (DNA)	4.9	65.0	1.3	0.017	-
2 (Histone complex)	9.3	151	7.3	0.094	-
3 (Nucleosome)	11.0	219	89.5	1.154	0.969
4 (Aggregate)	14.4	329	1.9	0.025	0.031

j: number of the component, $s_{20,\mathrm{w},j}$: sedimentation coefficient, M_j: molecular weight, w_j: weight fraction, c_j: mass concentration, t_j: contribution ratio in the forward scattering to the forward scattering intensity which eliminated liberated DNA and histone complex components ($ci_{\mathrm{exp}}(0) - c_{\mathrm{DNA}}i_{\mathrm{DNA}}(0) + c_{\mathrm{His}}i_{\mathrm{His}}(0) = c_{\mathrm{Nuc}}i_{\mathrm{Nuc}}(0) + c_{\mathrm{a}}i_{\mathrm{a}}(0)$).

Supplementary Table 7. Structural parameters of the nucleosome obtained by non-treated SAXS, AUC-SAS, and crystal structure.

	R_{gx} / Å	$i_x(0)$ / 10^{-1}mg^{-1}cm^2	M / kDa	χ^2
Non-treated SAXS	44.6 ± 0.2	2.04 ± 0.01	186 ± 1	1.20
AUC-SAS	44.7 ± 0.2	2.12 ± 0.01	193 ± 1	1.16
Crystal (3AFA[9])	38.6	2.18*	199*	-

R_{gx}: gyration radii (R_{ge}: non-treated SAXS, R_{g1}: others), $i_x(0)$: forward scattering intensities normalized by mass concentration, M : molecular weight calculated with $i_x(0)$[11]. χ^2: chi-squared value for the least square fitting with Guinier approximation.

*: Calculation with sequence including histone tails and remaining GSHM tags which are missing in 3AFA.

SUPPLEMENTARY NOTES

Supplementary Note 1. Forward scattering intensity of monomer.

The forward scattering intensity ratio t_k of the k-th component $c_k i_k(0)$ to the whole forward scattering $ci_{\mathrm{mul}}(0)$ is defined as

$$t_k \equiv \frac{c_k i_k(0)}{ci_{\mathrm{mul}}(0)}. \tag{S1}$$

Here, the forward scattering intensity of the j-th component $i_j(0)$ is represented as

$$i_j(0) = \frac{N_\mathrm{A}}{M_j} V_j^2 \Delta\rho_j^2 = \frac{N_\mathrm{A}}{M_j}\left(\frac{\bar{v}_j M_j}{N_\mathrm{A}}\right)^2 \Delta\rho_j^2 = \frac{\bar{v}_j^2 \Delta\rho_j^2 M_j}{N_\mathrm{A}} = \frac{m_j M_j}{N_\mathrm{A}}, \tag{S2}$$

where N_A, M_j, V_j, $\Delta\rho_j$, and $\bar{v}_j$ are Avogadro number, molecular weight, volume, scattering contrast, and partial specific volume for the j-th component, respectively[12], and j-th molecular scattering parameter m_j is defined as $m_j \equiv \bar{v}_j^2 \Delta\rho_j^2$. Therefore, eq.(S1) is rewritten with eq.(S2) as follows.

$$t_k = \frac{c_k i_k(0)}{\sum_{j=1}^{n} c_j i_j(0)} = \frac{c_k m_k M_k}{\sum_{j=1}^{n} c_j m_j M_j}. \tag{S3}$$

Assuming $\Delta\rho_j$ and $\bar{v}_j$ are identical for all components, all m_js are same: All components are proteins and their oligomers. When the j-th component is an oligomer with the aggregation number of j, $M_j = jM_1$, and then eq.(S3) is rewritten as follows.

$$t_k = \frac{c_k M_k}{\sum_{j=1}^{n} c_j M_j} = \frac{c_k k M_k}{\sum_{j=1}^{n} c_j j M_1} = \frac{c_k k}{\sum_{j=1}^{n} c_j j}. \tag{S4}$$

Thus, t_k can be calculated only with $\{c_j\}$, which is provided by AUC. This directly means that the forward scattering intensity $c_k i_k(0)$ can be calculated as $t_k ci_{\mathrm{mul}}(0)$ because $i_{\mathrm{mul}}(0)$ is provided as $i_{\mathrm{exp}}(0)$ with a SAS experiment for a multi-component system (example for BSA1 is listed in Supplementary Table 2).

Here, we should notice the relation between Guinier approximation and monodispersity again. As shown in Fig. 4, the observed $ci_{\mathrm{exp}}(q)$ follows the Guinier approximation and gave R_{ge} and $ci_{\mathrm{exp}}(0)$: It seems that this sample could be monodispersed. However, AUC clearly reveals that a few % of aggregates (5.9 % in the example, BSA1) generates an excess scattering $c_\mathrm{a} i_\mathrm{a}(0)$ ($= \sum_{j=2}^{4} c_j i_j(0)$ in the example) (red bar in Fig.4) in the observed whole $ci_{\mathrm{exp}}(0)$. In other words, Fig.4 shows that this sample includes the aggregates and also establishment of Guinier approximation does not stand for the monodispersity of the sample.

Supplementary Note 2. Scattering intensity in the high q-range.

2-1. Scattering intensity ratio of all oligomers to monomer, $r(q)$.

To estimate the effect of scattering intensities of oligomers (and monomer) on the whole scattering intensity, the intensity ratio $r(q)$ of whole scattering intensity $i_{\mathrm{mul}}(q)$ to that of monomer $i_1(q)$ is defined as follows.

$$r(q) \equiv \frac{i_{\mathrm{mul}}(q)}{i_1(q)} = \frac{\left(\sum_{j=1}^{n} c_j i_j(q)\right)/c}{i_1(q)}, \tag{S5}$$

Here, to calculate the scattering intensity of oligomers $i_j(q)$ ($j \geq 2$), the orientation between monomers in an oligomer is approximated to be averaged, and then the average monomer residential radius R, as shown in Supplementary Fig.3(a), is introduced as $R = \sqrt{5/3}R_{\mathrm{g1}}$: R_{g1} is the gyration radius of the monomer. Based on this assumption, two series of oligomers with different monomer configurations are built as examples: linearly aligned oligomers (Model 1) and closed packing oligomers (Model 2) shown in Supplementary Fig.3 (b) and (c). The scattering intensity of j-th oligomer $i_j(q)$ is represented with Debye function with $i_1(q)$.

$$i_j(q) = \frac{M_1}{M_j} i_1(q) \sum_{l=1}^{j} \sum_{m=1}^{j} \frac{\sin qL_{lm}}{qL_{lm}}, \tag{S6}$$

where L_{lm} ($= |\boldsymbol{L_{lm}}|$) is the distance between the centers of gravities of l-th and m-th monomers (Supplementary Fig.3(a)), and M_1 and M_j are the molecular weights of monomer and j-th oligomer, respectively. Accordingly, the scattering intensities $i_j(q)$ ($2 \leq j \leq 4$) and $r(q)$ are given for two models as follows.

[Model 1: Linear aligned oligomer model]

<u>Dimer (j = 2):</u>

$$i_2(q) = \frac{1}{2} i_1(q) \left(2 + \frac{2\sin qL}{qL}\right). \tag{S7}$$

$L_{l\,l+1} = L_{l+1\,l} = L = 2R.$

<u>Trimer (j = 3):</u>

$$i_3(q) = \frac{1}{3} i_1(q) \left(3 + \frac{4\sin qL}{qL} + \frac{2\sin 2qL}{2qL}\right). \tag{S8}$$

$L_{l\,l+2} = L_{l+2\,l} = 2L = 4R.$

Tetramer ($j = 4$):

$$i_4(q) = \frac{1}{4} i_1(q) \left(4 + \frac{6 \sin qL}{qL} + \frac{4 \sin 2qL}{2qL} + \frac{2 \sin 3qL}{3qL}\right). \tag{S9}$$

$L_{l\,l+3} = L_{l+3\,l} = 3L = 6R.$

$r(q)$:

$$r(q) = \frac{c_1}{c} + \frac{c_2}{2c}\left(2 + \frac{2\sin qL}{qL}\right) + \frac{c_3}{3c}\left(3 + \frac{4 \sin qL}{qL} + \frac{2 \sin 2qL}{2qL}\right)$$
$$+ \frac{c_4}{4c}\left(4 + \frac{6 \sin qL}{qL} + \frac{4 \sin 2qL}{2qL} + \frac{2 \sin 3qL}{3qL}\right). \tag{S10}$$

[Model2: Closed packing oligomer model]

Dimer ($j = 2$):

$$i_2(q) = \frac{1}{2} i_1(q) \left(2 + \frac{2 \sin qL}{qL}\right). \tag{S7}$$

$L_{l\,l+1} = L_{l+1\,l} = L = 2R.$

There is no difference between two models (Supplementary Fig.3(b) and (c)).

Trimer ($j = 3$):

$$i_3(q) = \frac{1}{3} i_1(q) \left(3 + \frac{6 \sin qL}{qL}\right). \tag{S11}$$

$L_{lm} = L = 2R.$

Tetramer ($j = 4$):

$$i_4(q) = \frac{1}{4} i_1(q) \left(4 + \frac{12 \sin qL}{qL}\right). \tag{S12}$$

$L_{lm} = L = 2R.$

$r(q)$:

$$r(q) = \frac{c_1}{c} + \frac{c_2}{2c}\left(2 + \frac{2 \sin qL}{qL}\right) + \frac{c_3}{3c}\left(3 + \frac{6 \sin qL}{qL}\right) + \frac{c_4}{4c}\left(4 + \frac{12 \sin qL}{qL}\right) \tag{S13}$$

2-2. Estimation of $r(q)$ in the higher q range: Demonstrated BSA1 solution.

As described in the main text, the scattering intensity of monomer in the high q range, $i_{1\mathrm{h}}(q_\mathrm{h})$, is approximated to be identical to that of oligomer $i_{j\mathrm{h}}(q_\mathrm{h})$ $(j \geq 2)$ (eq.(3)).

$$i_{1\mathrm{h}}(q_\mathrm{h}) \approx i_{\mathrm{mul}}(q_\mathrm{h}) = i_{\mathrm{exp}}(q_\mathrm{h})\,. \tag{3}$$

To figure out the range of this approximation for a highly-purified sample such as demonstrated BSA1 solution, $r(q)$ for two models were calculated by using the

observed $\{c_j\}$. Supplementary Fig.4 shows $r(q)$s for both models. Both $r(q)$s exhibit rapidly asymptotical approach to unity, and the deviation from unity is less than 1.8 % in $q^* R_{\mathrm{g1}} \geq 1.0$, where $R_{\mathrm{g1}}$is the gyration radius of the monomer. In other words, the error originated from the oligomer in the approximation eq.(3) is suppressed less than 1.8% in high q range $(q_{\mathrm{h}} \geq q^*)$. Therefore, in the case with a few % of aggregates (5.9 % in the example, BSA1), it can be approximated to be $i_{\mathrm{1h}}(q_{\mathrm{h}}) \approx i_{\mathrm{mul}}(q_{\mathrm{h}}) = i_{\mathrm{exp}}(q_{\mathrm{h}})$ in $q_{\mathrm{h}} > q^* = 1.0/R_{\mathrm{g1}}$. From empirical point of view, it has been known that the scattering intensity in the high q range is almost identical with that of monomer even though there are a few aggregates in the solution. Above calculation follows the empirical result. The limitation of the approximation is discussed in Supplementary Note 4-3.

Supplementary Note 3. Extraction of the monomer intensity $\boldsymbol{i_1(q)}$.

3-1. Set initial scattering intensity in the low $\boldsymbol{q}$-range $\boldsymbol{i_{\mathrm{1l}}(q_{\mathrm{l}})^*}$.

Since the initial scattering intensity in the low q-range, $i_{\mathrm{1l}}(q_{\mathrm{l}})^*$, should smoothly connect to the scattering intensity in the high q-range, $i_{\mathrm{1h}}(q_{\mathrm{h}})^*$, $i_{\mathrm{1l}}(q_{\mathrm{l}})^*$ is satisfied with $d\ln(i_{\mathrm{1l}}(q)^*)/dq^2 = d\ln(i_{\mathrm{1h}}(q)^*)/dq^2$ at the connection point q_{c}. Firstly, $i_{\mathrm{1h}}(q)^*$ is extrapolated into the lower q-range ($i_{\mathrm{1h}}(q_{\mathrm{l}})^* \approx i_{\mathrm{exp}}(q_{\mathrm{l}})$) as shown with open blue circles in Supplementary Fig.5(a). Considering Guinier approximation (eq.(5)), the correct $i_{\mathrm{1l}}(q_{\mathrm{l}})^*$ should be one of the straight lines connecting the point of the forward scattering intensity $i_1(0)$ (blue square in Supplementary Fig.5(a)) to the points on the extrapolated $i_{\mathrm{1h}}(q_{\mathrm{l}})^*$ (closed red and blue circles in Supplementary Fig.5(a)): The several candidates are shown with the red lines in Supplementary Fig.5(a). Supplementary Fig.5(b) shows the slopes for the candidates of $\ln(i_{\mathrm{1l}}(q_{\mathrm{l}})^*)$ and the extrapolated $\ln(i_{\mathrm{1h}}(q_{\mathrm{l}})^*)$ as a function of the connecting point q_c^2 (red and black lines in Supplementary Fig.5(b)), respectively. The cross point between two lines should be satisfied with $d\ln(i_{\mathrm{1l}}(q_{\mathrm{c}})^*)/dq^2 = d\ln(i_{\mathrm{1h}}(q_{\mathrm{c}})^*)/dq^2$ where $i_{\mathrm{1l}}(q_{\mathrm{l}})^*$ smoothly connects to $i_{\mathrm{1h}}(q_{\mathrm{h}})^*$: In the demonstrated BSA1 sample, the smoothly connected $i_{\mathrm{1l}}(q_{\mathrm{l}})^*$ is expressed with a blue line in Supplementary Fig.5(a) and the cross point is $q_{\mathrm{c}}^2 = 0.0053\ \text{Å}^{-2}$ (closed blue circle in Supplementary Fig.5(a)).

3-2. Expanded Guinier formula.

To expand the Guinier formula to the slightly higher q-range, it is necessary to add the Debye correction term $D_{\mathrm{c}}(q)$.

$$I(q) = I(0)\exp\left(-\frac{R_\mathrm{g}^2}{3}q^2\right) + D_\mathrm{c}(q) \tag{S14}$$

Here, we consider $D_\mathrm{c}(q)$ as follows. Scattering intensity with N atoms in solution is expressed by Debye formula[13]

$$I(q) = \sum_{l=1}^{N}\sum_{m=1}^{N} b_l b_m \frac{\sin(qr_{lm})}{qr_{lm}}, \quad (r_{lm} = |\mathbf{r}_l - \mathbf{r}_m|) \tag{S15}$$

where b_l and $\mathbf{r}_l$ are scattering contrast and coordinate of the l-th atom, respectively. As the approximation to the relatively higher q-range (more than Guinier range), its polynomial expansion is written as,

$$I(q) = \sum_{l=1}^{N}\sum_{m=1}^{N} b_l b_m \sum_{p=0}^{X} (-1)^p \frac{(qr_{lm})^{2p}}{(2p+1)!}$$

$$\cong I(0)\left[1 - \frac{R_\mathrm{g}^2}{3}q^2 + \sum_{p=2}^{X}(-1)^p \frac{r_{lm}^{2p}}{(2p+1)!}q^{2p}\right], \tag{S16}$$

where $I(0)$ and R_g are as follows.

$$I(0) = \sum_{l=1}^{N}\sum_{m=1}^{N} b_l b_m, \quad R_\mathrm{g}^2 = \frac{\sum_{l=1}^{N}\sum_{m=1}^{N} b_l b_m r_{lm}^2}{2\sum_{l=1}^{N}\sum_{m=1}^{N} b_l b_m}. \tag{S17}$$

On the other hand, the polynomial expansion of Guinier formula $I_\mathrm{G}(q)$ is expressed as

$$I_\mathrm{G}(q) = I(0)\exp\left(-\frac{R_\mathrm{g}^2}{3}q^2\right) \cong I(0)\left(1 - \frac{R_\mathrm{g}^2}{3}q^2 + \sum_{p=2}^{X}\left(-\frac{R_\mathrm{g}^2}{3}\right)^p \frac{q^{2p}}{p!}\right). \tag{S18}$$

Comparing eqs.(S16) with (S18), $D_\mathrm{c}(q)$, Debye correction term, is defined as the difference between these formula in the higher than q^4-term, i.e., $D_\mathrm{c}(q)$ is as follows.

$$D_\mathrm{c}(q) = \sum_{p=2}^{X}(-1)^p \frac{r_{lm}^{2p}}{(2p+1)!}q^{2p} - \sum_{p=2}^{X}\left(-\frac{R_\mathrm{g}^2}{3}\right)^p \frac{1}{p!}q^{2p} \equiv \sum_{p=2}^{X} u_p q^{2p}. \tag{S19}$$

Thus, the expanded Guinier formula is expressed as follows.

$$I(q) = I(0)\exp\left(-\frac{R_\mathrm{g}^2}{3}q^2\right) + \sum_{p=2}^{X} u_p q^{2p}. \tag{S20}$$

The expanded Guinier formula (eq.(S20)) is examined with the experiment data $ci_{\mathrm{exp}}(q)$ (Supplementary Fig.6). As the examination, the least square fitting of $ci_{\mathrm{exp}}(q)$ was performed with the expanded Guinier formula (eq.(S20)) by changing the highest order of $D_{\mathrm{c}}(q)$ in the q range over than the Guinier range ($q < 2.8/R_{\mathrm{g}}$) and then the obtained R_{g} was estimated. Independent upon q^2-order of $D_{\mathrm{c}}(q)$, the least square fittings were well-converged, and, as an example, Supplementary Fig.6(a) shows the result of the least square fitting with q^8-order (X = 4) of $D_{\mathrm{c}}(q)$, which well reproduced the experimental $ci_{\mathrm{exp}}(q)$ in the slightly higher q-range. Supplementary Fig.6(b) shows the obtained R_{g} as a function of q^2-order of $D_{\mathrm{c}}(q)$. It means that, by adding q^8-order of $D_{\mathrm{c}}(q)$ as the correction term, the expanded Guinier formula well-reproduces the experimental scattering intensity with holding the reasonable R_{g} value. It is also worth to note that, without Debye correction term, the R_{g} obtained with the simple Guinier formula in the wider q-range is found to be smaller than the reasonable one as shown in a red circle of Supplementary Fig.6(b).

3-3. Refinement of $i_1(q)^*$ with the expanded Guinier formula.

We obtained initial $i_1(q)^*$ by the connection of $i_{1\mathrm{l}}(q_{\mathrm{l}})^*$ given with Guinier formula to $i_{1\mathrm{h}}(q_{\mathrm{h}})^*$ obtained with the approximation $i_{1\mathrm{h}}(q_{\mathrm{h}}) \approx i_{\mathrm{exp}}(q_{\mathrm{h}})$ at q_{c} (Supplementary Note 2, Supplementary Fig.5). Here, $i_1(q)^*$ should be refined by the fitting with the expanded Guinier formula (Supplementary Note 3-2). The upper $q^2 (\equiv q_s^2)$ in the fitting was selected to be high enough, covering the twice of the connection point ($q_s^2 \geq 2q_{\mathrm{c}}^2$) and satisfying the range where the deviation of $r(q)$ from unity is less than 1% ($q_{\mathrm{s}}^2 = 2.8/R_{\mathrm{g1}}^2$ for BSA1).

Supplementary Fig.7 shows the $i_1(q)^*$ and the least square fitting with the expanded Guinier formula with q^8-ordered correction term $D_{\mathrm{c}}(q)$ in $q^2 \leq \left(2.8/R_{\mathrm{g1}}\right)^2$ for the BSA1 sample. Finally, the full $i_1(q)$ is provided by the connection of the expanded Guinier formula ($q^2 < q_s^2$) to $i_1(q)^*$ ($q^2 \geq q_s^2$).

Supplementary Note 4. Limitation and improvement of AUC-SAS.

The present AUC-SAS protocol is applicable to a solution with a small amount of aggregates. Here, it is worth to examine the upper limitation of the concertation of contaminated aggregates for the present AUC-SAS protocol and to discuss the possible improvement on the AUC-SAS to apply to the over limitation.

4-1. Applicable boundary in oligomer concentration.

AUC-SAS was examined with three BSA solutions containing various amounts of aggregates: BSA2-4 include with the different weight fractions of aggregates w_{agg} = 3.7 % (monomer: 96.3 %, dimer: 2.9 %, trimer: 0.5 %, tetramer: 0.3 %), w_{agg} = 12.4 % (monomer: 87.6 %, dimer: 10.4 %, trimer: 1.3 %, tetramer: 0.7 %), and w_{agg} = 18.6 % (monomer: 81.4 %, dimer: 16.0 %, trimer: 1.5 %, tetramer: 1.1 %), respectively. Their AUC spectra are shown in Supplementary Fig.11(a). Supplementary Fig.11 (b)-(d) shows their scattering intensities of monomers $i_1(q)$ provided with AUC-SAS (closed blue circles) and their Guinier plots. The present AUC-SAS protocol succeeded to provide the correct scattering intensities of the monomer for BSA2 and 3 but failed for BSA4. Therefore, the present procedure is applicable up to the solution with w_{agg}= 12.4% at least and it is supposed that the concentration boundary could locate around w_{agg}= 15%.

4-2. Possible improvement for higher concentration of aggregates.

Let us consider the possible improvement. As shown in Supplementary Fig.11(d), the experimental scattering intensity of BSA4 did not hold Guinier approximation. Therefore, the failure of the present protocol would arise from the inaccuracy of $i_{\mathrm{exp}}(0)$, which causes the wrong $i_1(0)$. As an improved approach, $i_1(0)$ should be directly calculated with the following equation[12] instead of $t_1 c i_{\mathrm{exp}}(0)$ (Supplementary Note 1).

$$i_1(0) = M N_{\mathrm{A}}^{-1} \left(\rho_{\mathrm{p}} - \rho_{\mathrm{s}} \bar{v}\right)^2 r_0^2, \quad \text{(S21)}$$

where M, N_{A}, ρ_{p}, ρ_{s}, $\bar{v}$, and r_0 are the molecular weight, Avogadro number, number of electrons per mass of protein, number of electrons per volume of solvent, partial specific volume, and scattering length of an electron, respectively. The calculated $i_1(0)$s with the improved approach and present methods are indicated with the closed blue and cyan squares in right figures (Guinier plot) in Supplementary Fig.12, respectively. There is no difference in $i_1(0)$s calculated with two methods in BSA3 sample whereas the calculated $i_1(0)$s with the improved approach is smaller than that with the present AUC-SAS protocol ($t_1 c i_{\mathrm{exp}}(0)$) in BSA4 sample. Applying the procedures, steps 4 and 5A, a new $i_1(q)$s are also provided as shown in blue closed circles of Supplementary Fig.12. The improved approach led $i_1(q)$s consistent with those of SEC-SAXS even with 18.6 % of weight fraction of aggregates.

4-3. Effect of $r(q)$ as a function of concentration of oligomers.

The approximation $i_{1\mathrm{h}}(q_{\mathrm{h}}) \approx i_{\mathrm{mul}}(q_{\mathrm{h}})$ (eq.(3)) holds in the higher q range when the oscillation amplitude of $r(q)$, Amp_r, is negligibly small: As described in the previous

section, the present protocol succeeds for the sample with the low amount of aggregates (< 12.4 %). Here, Amp_r should be discussed as a function of concentration of oligomers to estimate the boundary value of Amp_r.

For a simple discussion to estimate the maximum $Amp\_r_{\max}$, all aggregates supposed to be dimers. In this case, $|1 - r(q)|$ in the higher q range becomes the maximum at $qR_{g1} = 1.7$ (Supplementary Fig.13(a)) and then $Amp\_r_{\max}$ can be calculated using eq.(S10) with $c_3 = c_4 = 0$ as follows.

$$Amp\_r_{\max} = |1 - r(q)|_{q=1.7/R_{g1}} = \frac{0.216\, c_2}{c} \quad \text{(S22)}$$

Supplementary Fig.13(b) shows $Amp\_r_{\max}$ in c_2/c from 0 to 25. The applicable boundary range (12.4 % -18.6 %) described in Supplementary Note 4-1 is drawn with gray color, meaning that $Amp\_r_{\max}$ should be less than ca. 3% for application of the present AUC-SAS protocol.

Supplementary Note 5. SV-AUC for the fast association-dissociation system.

Firstly, the complex formation in the demonstrated system was examined with SV-AUC method because it could not be confirmed by SEC method (Supplementary Fig.14). Two buffers were used for this purpose; one was Phosphate Buffered Saline (PBS) consisting of 8.1 mM Na_2HPO_4, 1.47 mM KH_2PO_4, 137 mM NaCl, and 2.7mM KCl (pH 7.4), and the other was 10mM sodium phosphate buffer (PB) (pH 7.0). As shown in Supplementary Fig.16(a), the complex formation was not observed in PBS because the observed peak positions of the mixture solution were completely same as those of individual components. On the other hand, as shown in Supplementary Fig.16(b), two peaks in the mixture with 10 mM PB were observed at the different positions from those of individual components, meaning not only the complex formation but also existence of the fast association-dissociation[14].

In conclusion, the difference between two buffers indicates that the driving force of the complex formation is an ion interaction between the proteins: the complex is formed in the buffer without salt (10 mM PB), while is not in PBS which contains salts. However, practically speaking, it is difficult to find the concentration distribution of all component with SV-AUC data because the observed peaks did not correspond to individual components but to the intermediates states. Therefore, to find the concentration distribution of all components, we conducted a SE-AUC method.

Supplementary Note 6. SE-AUC for determination of K_D and concentration of each component.

SE-AUC measurement was conducted for mixture solution of hHR23b-UBL and PNGase-PUB in 10mM PB (pH7.0) at three concentrations ([hHR23b-UBL] = [PNGase-PUB] = 100 μM, 75 μM, and 50 μM) and three rotor speeds (20000, 30000, and 35000 rpm) (Supplementary Fig.17). The fitting analysis was carried out with the association-dissociation equilibrium model as shown in eq.(7) (see METHODS). For accurate determination of the free parameters, the global fitting analysis was carried out for the three different concentrations and three different rotor speeds with K_D as a parameter. As the result of fitting analysis, $K_D = 227 \pm 28$ μM was given. Consequently, the concentration of each component in the solution was revealed to be $c_A = c_B = 75 \pm 3$ μM and $c_{AB} = 25 \pm 3$ μM at the feed concentrations for SAXS measurement ([hHR23b-UBL] = [PNGase-PUB] = 100 μM).

Supplementary Note 7. Structural analysis of the complex of hHR23b-UBL and PNGase-PUB.

The extracted $i_{AB}(q)$ is shown in Supplementary Fig.18(a), and the gyration radius and forward scattering intensity are also listed in Supplementary Table 4. In addition, the three-dimensional model analysis was conducted as follows. The initial model (Model 1 in Supplementary Fig.18(c)) was constructed by docking simulation program to face the interaction surfaces, which were revealed by NMR measurements[2] (see detail in the reference). The SAXS intensity calculated from Model 1 is shown with a red curve in Supplementary Fig.18(a), which deviates from the experimental $i_{AB}(q)$ in $q \geq 0.15$ Å^{-1} and results in the large χ^2 = 10.4. For refining the structure, the normal mode analysis (NMA) was carried out with Pepsi-SAXS-NMA[3,4] on Model 1. The refined model (Model 2) are shown in Supplementary Fig.18(c) and its scattering intensity (the orange curve in Supplementary Fig.18(a)) reduced χ^2 (= 2.8). Compared with Models 1 and 2, the boundary structure between two domains were preserved while it is suggested that the loop or terminal part could be deformed in Model 2. Thus, AUC-SAS offered more reasonable structure taking into account the structural fluctuation in solution.

Supplementary Note 8. Application to the complicated multi-component system.

As a demonstration for a more complicated example, AUC-SAS was applied to the multi-component system involving the concerned complex, dissociated components, and aggregates. Supplementary Fig.19 shows the result of AUC-SAS for the nucleosome solution (see METHODS). SV-AUC (Supplementary Fig.19(a) and Supplementary Table 6) revealed that the solution contained the nucleosome as a major component (concerned complex; *j*=3) as well as minor components, liberated DNA (*j*=1), liberated histone complex (*j*=2), and aggregates (*j*=4). Since the scattering from the solution is affected by the all components, the intensity is represented as

$$I_{\mathrm{exp}}(q) = ci_{\mathrm{exp}}(q) = c_{\mathrm{DNA}}i_{\mathrm{DNA}}(q) + c_{\mathrm{His}}i_{\mathrm{His}}(q) + c_{\mathrm{Nuc}}i_{\mathrm{Nuc}}(q) + c_{\mathrm{a}}i_{\mathrm{a}}(q), \quad \text{(S23)}$$

where the subscripts represent liberated DNA, liberated histone complex, the nucleosome, and aggregates, respectively. Firstly, the contribution of liberated DNA ($c_{\mathrm{DNA}}i_{\mathrm{DNA}}(q)$) and histone complex ($c_{\mathrm{His}}i_{\mathrm{His}}(q)$) were eliminated from $ci_{\mathrm{exp}}(q)$ with "*Complex Scattering Extraction*" procedure. The scattering intensity of the liberated DNA was measured with for the solo DNA solution and that of the liberated histone complex was calculated from the part of the crystal structure (PDB code 3AFA[9]). Subsequently, the aggregation term ($c_{\mathrm{a}}i_{\mathrm{a}}(q)$) was removed with "*Removing Aggregation*" procedure and then the precise scattering intensity of the nucleosome ($c_{\mathrm{Nuc}}i_{\mathrm{Nuc}}(q)$) was obtained (Supplementary Fig.19(b)-(c)). Here, t_k was calculated for nucleosome and aggregation components in$c_{\mathrm{Nuc}}i_{\mathrm{Nuc}}(0) + c_{\mathrm{a}}i_{\mathrm{a}}(0)$ from eq.(S3) with $\bar{v}_{\mathrm{Nuc}} = 0.669\ \mathrm{cm^3\ g^{-1}}$, $\bar{v}_{\mathrm{a}} = 0.696\ \mathrm{cm^3\ g^{-1}}$, $\Delta\rho_{\mathrm{Nuc}} = 474\ \mathrm{e\ nm^{-3}}$, $\Delta\rho_{\mathrm{a}} = 458\ \mathrm{e\ nm^{-3}}$, $M_{\mathrm{Nuc}} = 219\ \mathrm{kDa}$, and $M_{\mathrm{a}} = 329\ \mathrm{kDa}$, considering that the scattering power between DNA and protein are different.

REFERENCES

1 Inoue, R. *et al.* Newly developed Laboratory-based Size exclusion chromatography Small-angle x-ray scattering System (La-SSS). *Scientific Reports* **9**, 1-12 (2019).

2 Kamiya, Y. *et al.* NMR characterization of the interaction between the PUB domain of peptide: N-glycanase and ubiquitin-like domain of HR23. *FEBS letters* **586**, 1141-1146 (2012).

3 https://files.inria.fr/NanoDFiles/Website/Software/Pepsi-SAXS/MacOS/Pepsi-SAXS-NMA.

4 Grudinin, S., Garkavenko, M. & Kazennov, A. Pepsi‐SAXS: an adaptive method for rapid and accurate computation of small‐angle X‐ray scattering profiles. *Acta Crystallographica Section D* **73**, 449-464 (2017).

5 Svergun, D. I. Restoring low resolution structure of biological macromolecules from solution scattering using simulated annealing. *Biophysical journal* **76**, 2879-2886 (1999).

6 Svergun, D. I. Determination of the regularization parameter in indirect-transform methods using perceptual criteria. *J Appl Crystallogr* **25**, 495-503 (1992).

7 Bujacz, A. Structures of bovine, equine and leporine serum albumin. *Acta Crystallographica Section D: Biological Crystallography* **68**, 1278-1289 (2012).

8 Stein, P. E., Leslie, A. G., Finch, J. T. & Carrell, R. W. Crystal structure of uncleaved ovalbumin at 1.95 Å resolution. *Journal of molecular biology* **221**, 941-959 (1991).

9 Tachiwana, H. *et al.* Structural basis of instability of the nucleosome containing a testis-specific histone variant, human H3T. *Proceedings of the National Academy of Sciences* **107**, 10454-10459 (2010).

10 Russo, C. J. & Passmore, L. A. Ultrastable gold substrates for electron cryomicroscopy. *Science* **346**, 1377-1380 (2014).

11 Mylonas, E. & Svergun, D. I. Accuracy of molecular mass determination of proteins in solution by small-angle X-ray scattering. *Applied Crystallography* **40**, 245-249 (2007).

12 Trewhella, J. *et al.* 2017 publication guidelines for structural modelling of small-angle scattering data from biomolecules in solution: an update. *Acta Crystallographica Section D: Structural Biology* **73** (2017).

13 Feigin, L. & Svergun, D. *Structure Analysis by Small-Angle X-Ray and Neutron Scattering*. (New York: Plenum Press, 1987).

14 Dam, J., Velikovsky, C. A., Mariuzza, R. A., Urbanke, C. & Schuck, P. Sedimentation velocity analysis of heterogeneous protein-protein interactions: Lamm equation modeling and sedimentation coefficient distributions c(s). *Biophysical journal* **89**, 619-634 (2005).
