# Supplementary material for: Integral approach to biomacromolecular structure by analytical-ultracentrifugation and small-angle scattering
Source: Commun Biol. 2020 Jun 8;3:294. doi: 10.1038/s42003-020-1011-4 (PMC7280208; doi:10.1038/s42003-020-1011-4)
Supplement: Supplementary file 2 — Description of Additional Supplementary Files [file 42003_2020_1011_MOESM2_ESM.pdf]

Description of Additional Supplementary Files

File Name: Supplementary Data 1
Description: Data1.xls includes SAXS data of Fig.2. There are three data, $c^{*}i_{\mathrm{exp}}(q)$, $c_1{}^{*}i_1(q)$ and $c_a{}^{*}i_1(q)$. Each data has three columns, q, scattering int ensity and its errors.

File Name: Supplementary Data 2
Description: Data2.xls includes SV-AUC data of Fig.3. The data has two colu mns, $s_{20,\mathrm{w}}$ and $\mathrm{c}(s_{20,\mathrm{w}})$.

File Name: Supplementary Data 3
Description: Data3.xls includes SAXS data of Fig.6(a). There are four data, $c^{*}i_{\mathrm{exp}}(q)$, $c_A{}^{*}i_{\mathrm{A}}(q)$, $c_B{}^{*}i_{\mathrm{B}}(q)$ and $c_{AB}{}^{*}$ $i_{\mathrm{AB}}(q)$. Each data has three colu mns, q , scattering intensity and its errors.

File Name: Supplementary Data 4
Description: Data4.xls includes SE-AUC data of Fig.6(b). There are three d ata with rotation speeds of 20,000 rpm, 30,000rpm and 35,000 rpm. Each dat a has two columns, r and $a(r; K_d)$.
